# Activation of lung megakaryocyte **α**7 nAChR exacerbates allergic airway inflammation via IL-33/p38 MAPK signaling

**DOI:** 10.1172/jci.insight.202201

**Published:** 2026-09-08

**Authors:** Hang Wu, Rujia Tao, Shitao Xie, Yao Zhou, Jin-Fu Xu, Zhenwei Xia, Xiao Su

**Affiliations:** 1 Department of Pediatrics, Ruijin Hospital, Shanghai Jiao Tong University School of Medicine, Shanghai, China.; 2 Key Laboratory of Immune Response and Immunotherapy, Shanghai Institute of Materia Medica, Chinese Academy of Sciences, Shanghai, China.; 3 Department of Respiratory Medicine, Hunan Provincial People’s Hospital, The First Affiliated Hospital of Hunan Normal University, Changsha, China.; 4 Department of Respiratory and Critical Care Medicine, Tongji Hospital, School of Medicine, Tongji University, Shanghai, China.; 5 Shanghai Key Laboratory of Lung Inflammation and Injury, Department of Pulmonary Medicine, Zhongshan Hospital, Fudan University, Shanghai, China.

**Keywords:** Immunology, Inflammation, Pulmonology, Allergy, Asthma

## Abstract

The cholinergic antiinflammatory pathway attenuates lung inflammation via the α7 nicotinic acetylcholine receptor (α7 nAChR) on immune cells. However, the role of α7 nAChR on lung megakaryocytes (Mks) in allergic airway inflammation remains unknown. In this study, allergen-challenged mouse models were used with conditional Mk-specific *Chrna7* knockout, pharmacological activation (GTS-21), and Mk reconstitution. IL-33 expression and p38 MAPK signaling were assessed. We found that allergen challenge upregulated α7 nAChR specifically in lung Mks. Mk-specific *Chrna7* deletion significantly alleviated allergic airway inflammation, whereas GTS-21 exacerbated inflammation via an Mk-dependent mechanism. Reconstitution with α7 nAChR^+^ Mks restored airway inflammatory responses. Mechanistically, α7 nAChR activation promoted Mk IL-33 synthesis and secretion through p38 MAPK signaling. Taken together, our results show that α7 nAChR on lung Mks plays a proinflammatory role in allergic airway inflammation, challenging its classical antiinflammatory paradigm and revealing pathogenic mechanisms.

## Abstract 2

Activation of α7 nAChR on lung megakaryocytes exacerbates allergic airway inflammation via p38 MAPK/IL-33, revealing novel disease pathogenesis.

## Introduction

Allergic asthma is one of the most prevalent airway autoimmune diseases, involving multiple cells and cellular components, thus representing a heterogeneous disease with complex phenotypes and endotypes (1). The pathogenesis of asthma is multifaceted and has not been fully elucidated. It is now generally accepted that airway epithelial cells (AECs) (2), dendritic cells (DCs) (3), group 2 innate lymphoid cells (ILC2s), eosinophils (4), basophils (5), neutrophils (6), mast cells (7), macrophages (8), and other cells are involved in regulating asthma. In innate immune responses, ILC2s release a large amount of type 2 cytokines, including interleukin 4 (IL-4), IL-5, and IL-13, which promote airway inflammation by recruiting immune cells, such as eosinophils and neutrophils, and aggravating airway hyperresponsiveness (9). Type 2 cytokines can also be release by type 2 T helper (Th2) cells in adaptive immune responses (9, 10). In recent years, some studies have found that some rare cells, such as pulmonary neuroendocrine cells (PNECs), also play an important role in allergic asthma (11). However, whether other kinds of cells participate in allergic airway inflammation is still unknown.

Megakaryocytes (Mks) are precursor cells that produce platelets in the hematopoietic system (12), and they exist in other places such as the lung, liver, spleen, and peripheral blood (13). A recent study has found that the lung, which is an important organ for platelet production in the body, harbors a large number of Mks (14). Further research found that compared with bone marrow (BM) Mks, lung Mks express more immune-related molecules and had the ability to phagocytose and process ovalbumin (OVA) (15). Another study demonstrated that neutrophil “plucking” on Mks drives platelet production and boosts cardiovascular disease (16). IL-33 in platelets has been found to promote allergic airway inflammation (17), but its role within Mks themselves has not been addressed. Therefore, it is necessary to conduct further research into Mks’ function in airway inflammation.

The α7 nicotinic acetylcholine receptor (α7 nAChR) is composed of a homopentamer formed by 5 α7 subunits that exhibits a strong affinity for α-bungarotoxins (18). The cholinergic antiinflammatory pathway modulates inflammatory responses in macrophages and other immune cells through vagus nerve/α7 nAChR signaling (19). The α7 nAChR is expressed on various cells, including epithelial cells (20), macrophages (21), ILC2s (22), and T lymphocytes (23). In virus infection, it has been discovered that the activation of α7 nAChR on T cells promotes the replication of the human immunodeficiency virus (HIV) (24). However, the activation of α7 nAChR on alveolar macrophages suppresses the expression levels of tumor necrosis factor α (TNF-α) and IL-1β genes induced by lipopolysaccharide (LPS), thereby inhibiting the inflammatory cytokine storm (19, 25). Additionally, the release of acetylcholine by the vagus nerve activates α7 nAChR^+^CD11b^+^ cells, which promotes the phosphorylation of AKT1 to inhibit the mobilization and recruitment of inflammatory cells in the spleen to the lung (26). Furthermore, α7 nAChR on alveolar type II (AT2) cells modulates the repair of acute lung injury, and conditional deletion of *Chrna7* in AT2 cells impedes the lung repair process (27). Above all, these studies manifest that α7 nAChR plays an important role in infection and immunity. But whether α7 nAChR regulates lung Mks remains unknown.

IL-33 plays an important role in allergic airway inflammation (28). Endothelial cells, fibroblasts, and mucosal epithelial cells all exhibit high expression of IL-33 mRNA (29). As an alarmin molecule, IL-33 can be released extracellularly when cells are stimulated by allergens (30). However, the exact secretion mechanism of IL-33 remains incompletely understood. Recent studies have found that upon stimulation by enzymatic allergens, the p40 N-terminal form of gasdermin D promotes the secretion of IL-33 from the cytosol to the extracellular space (31). However, whether activation of α7 nAChR affects IL-33 expression and secretion and therefore influences allergic airway inflammation is unknown.

In this study, we investigated whether and how α7 nAChR modulates lung Mks during allergic airway inflammation. We demonstrated that lung Mks expressed higher levels of α7 nAChR than BM Mks, and that activation of α7 nAChR expressed on Mks exacerbated allergic airway inflammation. In addition, we found that activation of α7 nAChR promoted the expression and release of IL-33 in Mks, which was dependent on the p38 MAPK signaling pathway. These findings reveal how vagus nerve α7 nAChR signaling regulates lung Mks in allergic airway inflammation.

## Results

### Mks worsen allergic airway inflammation.

**Figure 1:**
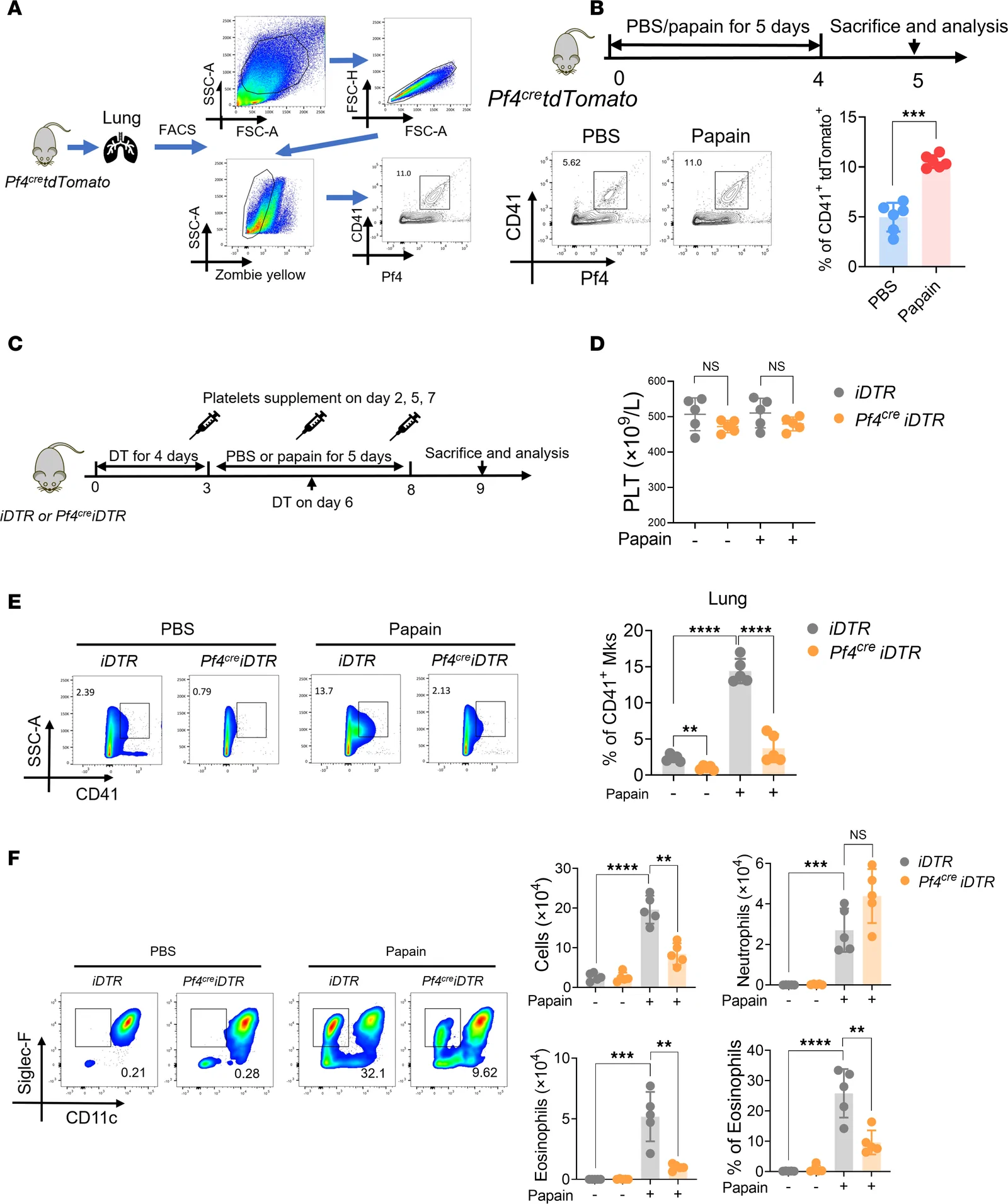
Mks participate in allergic airway inflammation. (**A**) The flow cytometric gating strategy for labeling lung Mks in *Pf4^cre^tdTomato* mice. (**B**) The percentages of Mks in papain and control groups. (**C**) Schematic diagram of Mk depletion and platelet supplementation in papain-induced allergic airway inflammation by using *Pf4^cre^iDTR* mice. (**D**) Determination of platelets in blood by peripheral blood test in each group. (**E**) The percentage of CD41^+^ cells in FSC-high/SSC-high lung cells between papain and control groups. (**F**) Determination of total cells and eosinophils (CD45^+^Siglec-F^+^CD11c^–^ cells) in BALF by flow cytometry. Data are representative of at least 3 independent experiments and are presented as mean ± SD (*n* = 5). **P < 0.01; ***P < 0.001; ****P < 0.0001 by 2-sided *t* test (**B**) or 1-way ANOVA with Tukey’s post hoc analysis (**D**–**F**). NS, not significant.

It has been reported that Mks and platelets play various important roles in many respiratory diseases, such as acute respiratory distress syndrome (32), pulmonary fibrosis (33), and chronic obstructive pulmonary diseases (34). Takeda et al. found that platelets can modulate eosinophilic airway inflammation (17). However, whether Mks themselves impact allergic airway inflammation is still unknown. In our study, we generated *Pf4^cre^tdTomato* mice to label Mks in lung. Then we challenged *Pf4^cre^tdTomato* mice with PBS or papain for 5 days via intratracheal instillation. The results showed that the proportions of tdTomato^+^ and CD41^+^ double-positive Mks in lung tissues of the papain group were significantly higher than those of the PBS group (Figure 1, A and B). Similar results were seen in ovalbumin-induced (OVA-induced) and house dust mite–induced (HDM-induced) allergic airway inflammation models; we found the percentage of lung Mks increased in the asthma group (Supplemental Figure 1A; supplemental material available online with this article; https://doi.org/10.1172/jci.insight.202201DS1).

Next, we generated *Pf4^cre^iDTR* mice to conditionally deplete Mks after diphtheria toxin (DT) injection (Figure 1C). To eliminate the potential impact of platelet depletion by DT injection, each *Pf4^cre^iDTR* mouse received donor platelets 3 times. Blood test verified the platelet replenishment was effective (Figure 1D). Flow cytometric analysis showed that the percentage of CD41^+^ Mks in FSC-high/SSC-high cells in lung was markedly decreased after DT injection in *Pf4^cre^iDTR* mice (Figure 1E), but the injection of DT did not affect the numbers of DCs, monocytes, neutrophils, CD4^+^ T cells, and CD8^+^ T cells in lung (Supplemental Figure 1, B and C). We also evaluated the impact of DT injection on myeloid cells and lymphocytes in the peripheral blood of *Pf4^cre^iDTR* mice. Our results indicated that under our experimental conditions, DT-treated *Pf4^cre^iDTR* mice exhibited no significant fluctuations in peripheral blood lymphocyte counts between day 0 and day 9 (Supplemental Figure 1D). Furthermore, no significant differences were observed in myeloid cells (such as neutrophils, monocytes, and eosinophils) across the groups (Supplemental Figure 1E). We found that depletion of Mks significantly relieved papain-induced airway inflammation, including the decreased total cell numbers in bronchoalveolar lavage fluid (BALF), eosinophil infiltration in BALF, and eosinophil and ILC2 numbers in lung. At the same time, it had no effect on the number of neutrophils in BALF (Figure 1F and Supplemental Figure 1F). We also examined the effect of Mk depletion on other important parameters in peripheral blood. We found Mk depletion did not affect the number of white blood cells, red blood cells (RBCs), hemoglobin, mean platelet volume, and mean corpuscular hemoglobin concentration in peripheral blood (Supplemental Figure 1G). These observations revealed that Mks participated in and accelerated the development of allergic airway inflammation, independently of platelets.

### Lung Mks express α7 nAChR.

**Figure 2:**
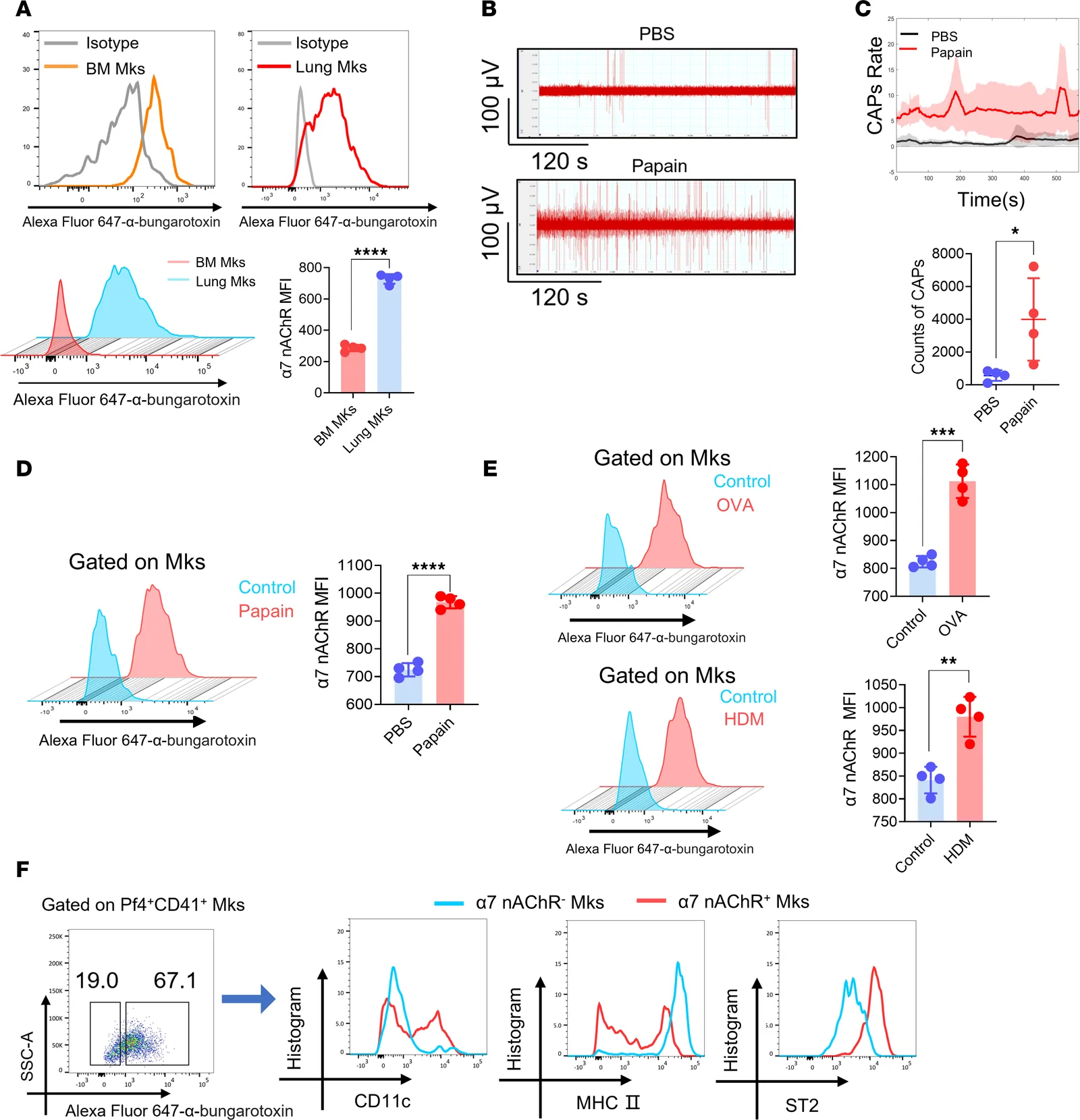
Lung Mks are modulated by vagus nerve/α7 nAChR axis. (**A**) The expression of α7 nicotinic acetylcholine receptor (α7 nAChR) on Mks from BM and lung using Alexa Fluor 647–conjugated α-bungarotoxin, a nicotinic acetylcholine receptor blocker. (**B**) Vagus nerve electrical activity after PBS and papain stimulation for 5 days in vivo. (**C**) Vagus nerve compound action potential (CAP) rate and CAP counts after PBS and papain stimulation for 5 days in vivo. (**D**) Upregulated expression of α7 nAChR on lung Mks after papain stimulation for 5 days in vivo. (**E**) Upregulated expression of α7 nAChR on lung Mks in OVA and HDM models in vivo. (**F**) The difference in CD11c, MHC-II, and ST2 expression between α7 nAChR^+^ and α7 nAChR^–^ lung Mks. Data are representative of at least 3 independent experiments and are presented as mean ± SD (*n* = 4). *P < 0.05, **P < 0.01, ***P < 0.001, ****P < 0.0001 by 2-sided *t* test (**A** and **C**–**E**). NS, not significant.

α7 nAChR is present on various cells, including T cells, macrophages, epithelial cells, and others (18, 23). Here, we investigated the expression of α7 nAChR on Mks. In *Pf4^cre^tdTomato* mice, we discovered that Mks from both the BM and lung expressed α7 nAChR; however, the expression of α7 nAChR on lung Mks was much greater than that on BM Mks (Figure 2A). Then, we tested the excitability of the vagus nerve in papain-induced allergic airway inflammation. We found that in papain-induced airway inflammation, the vagus nerve was activated (Figure 2B) and the number of compound action potentials (CAPs) was significantly increased (Figure 2C). Additionally, we found that the expression of α7 nAChR on lung Mks was upregulated in vivo after papain challenge for 5 days (Figure 2D). Similar results were observed in OVA- or HDM-induced airway inflammation (Figure 2E). These findings indicated that lung Mks expressed α7 nAChR, which could be regulated by vagal signals during allergic airway inflammation. Previous studies have shown that there are differences in the expression of immune molecules such as MHC-II, CD40, and CD11c between lung and BM Mks. In our study, we also found that CD11c and ST2 (IL-33 receptor) were expressed at high levels on α7 nAChR^+^ lung Mks relative to α7 nAChR^–^ lung Mks. However, MHC-II showed the opposite expression (Figure 2F), suggesting their different immune functions and timing. Most importantly, we confirmed that lung Mks express α7 nAChR at relatively high levels, and that α7 nAChR on lung Mks may play a potential role in allergic airway inflammation.

### Deletion of Chrna7 in Mks alleviates allergic airway inflammation.

**Figure 3:**
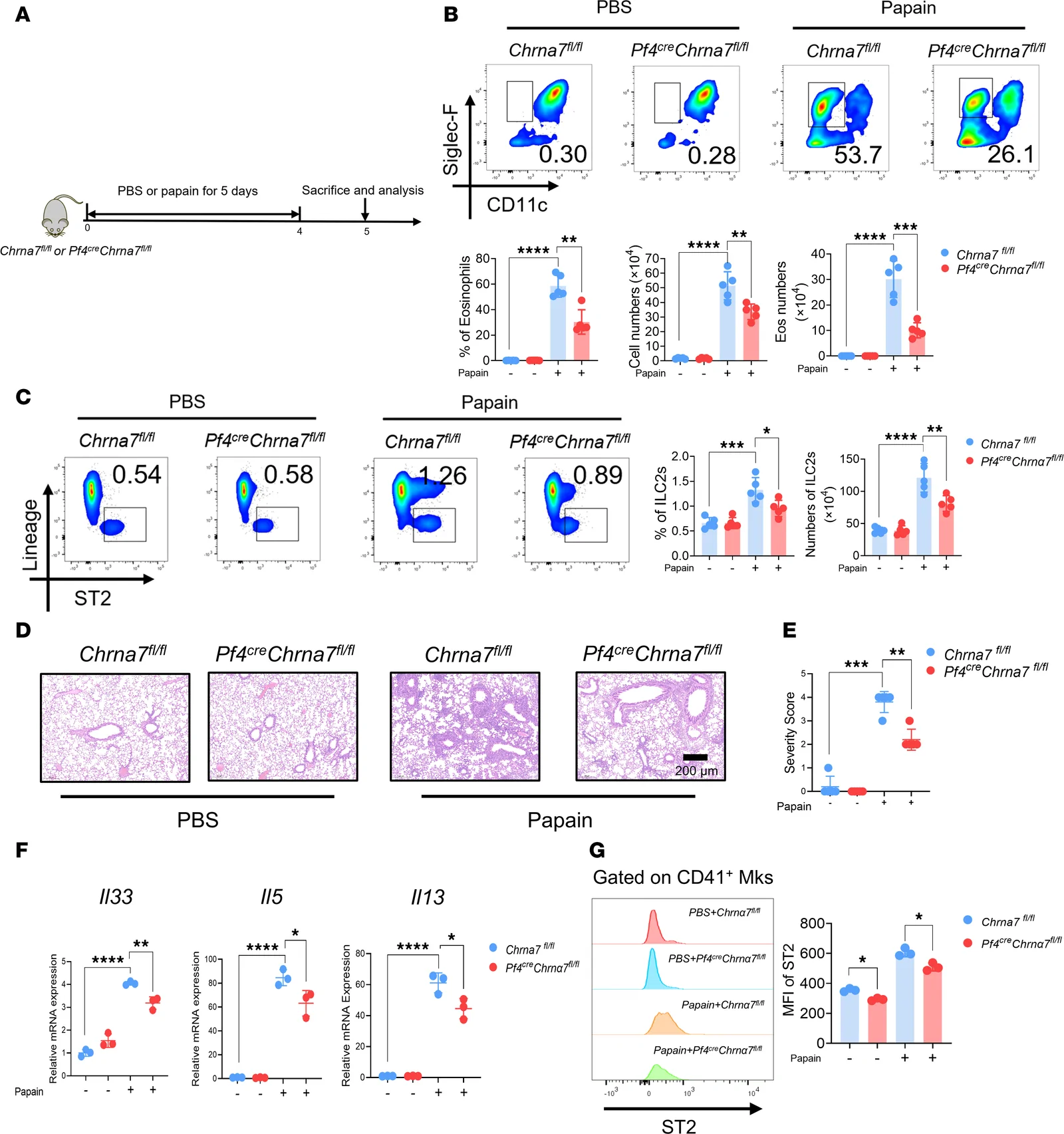
Deletion of α7 nAChR on Mks alleviates papain-induced allergic airway inflammation. (**A**) Schematic diagram of intratracheal PBS or papain delivery to *Chrna7^fl/fl^* and *Pf4^cre^Chrna7^fl/fl^* mice for 5 days. (**B**) Determination of total cells and eosinophils in BALF by flow cytometry. (**C**) Determination of ILC2s in lung tissue by flow cytometry. (**D**) Representative H&E staining of lung histology. Scale bar: 200 μm. (**E**) Severity score of lung tissue. (**F**) The relative mRNA expression of *Il33*, *Il5*, and *Il13* in lung tissue homogenate tested by RT-qPCR. (**G**) Expression of ST2 on Mks was tested by flow cytometry. Data are representative of at least 3 independent experiments and are presented as mean ± SD (*n* = 3–5). *P < 0.05; **P < 0.01; ***P < 0.001; ****P < 0.0001 by 1-way ANOVA with Tukey’s post hoc analysis (**B**, **C**, and **E**–**G**). NS, not significant.

**Figure 4:**
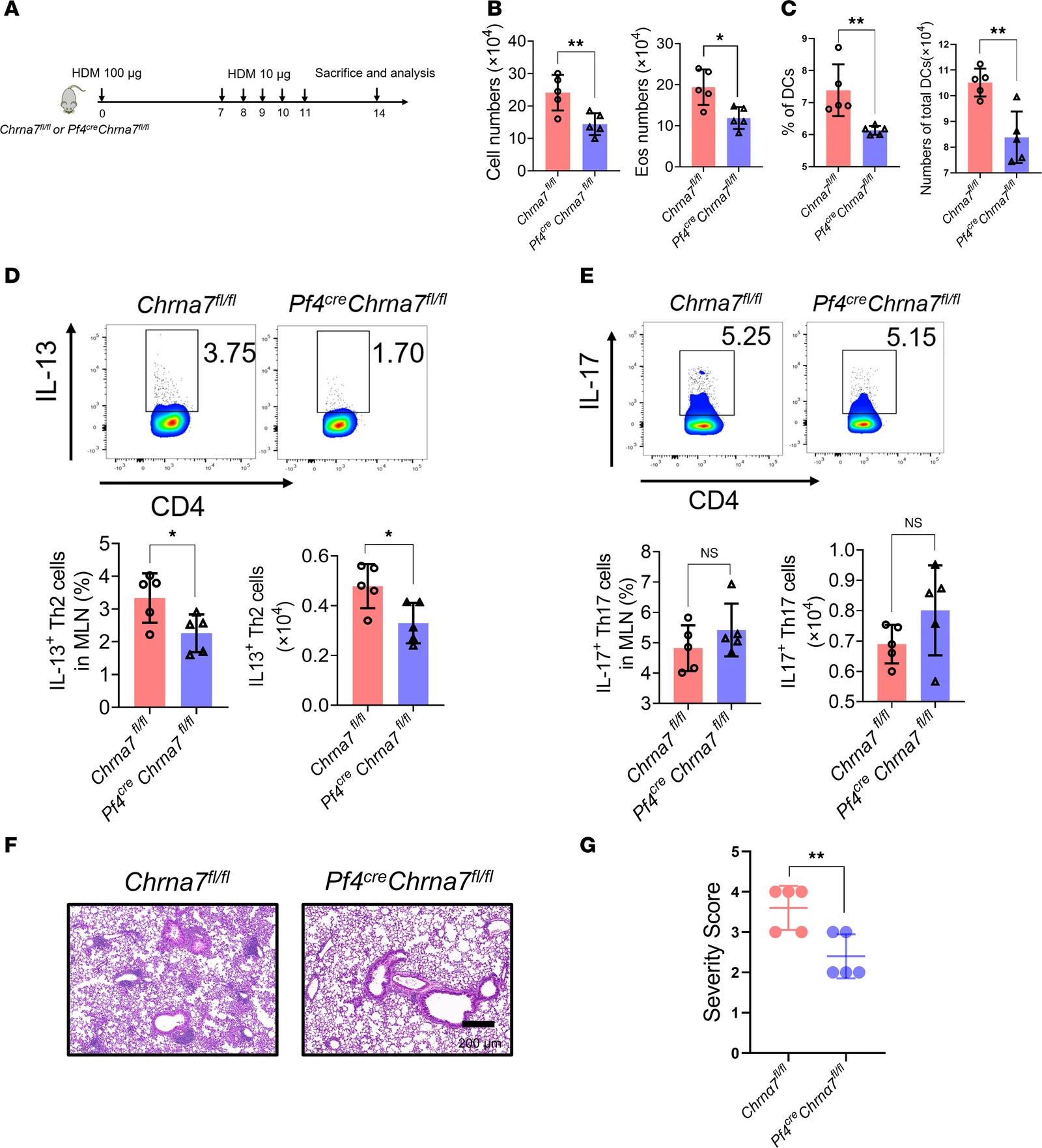
Deletion of α7 nAChR on Mks alleviates HDM-induced type 2 airway inflammation. (**A**) Schematic diagram of intratracheal HDM delivery to *Chrna7^fl/fl^* and *Pf4^cre^Chrna7^fl/fl^* mice for 14 days. (**B**) Total cells and eosinophils in BALF determined by flow cytometry. (**C**) Total DCs in lung determined by flow cytometry. (**D**) Th2 cells (CD4^+^IL-13^+^) in MLNs determined by flow cytometry. (**E**) Th17 cells (CD4^+^IL-17^+^) in MLNs determined by flow cytometry. (**F**) Representative H&E staining of lung tissue. Scale bar: 200 μm. (**G**) Severity score of lung tissue. Data are representative of at least 3 independent experiments and are presented as mean ± SD (*n* = 5). *P < 0.05, **P < 0.01 by 2-sided *t* test (**B**–**E**, and **G**). NS, not significant.

**Figure 5:**
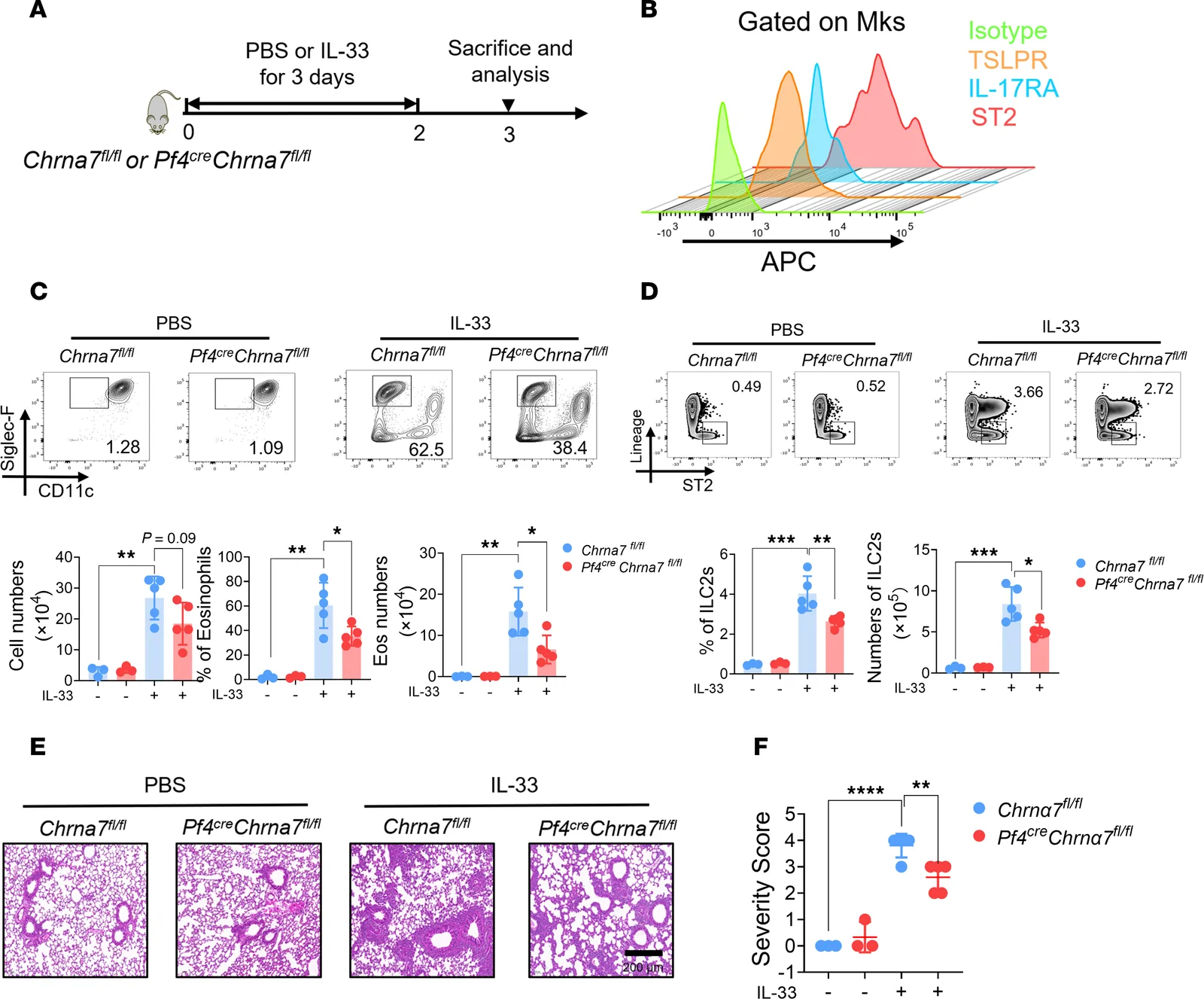
Deletion of α7 nAChR on Mks attenuates IL-33–induced airway inflammation. (**A**) Schematic diagram of intratracheal PBS or IL-33 delivery to *Chrna7^fl/fl^* and *Pf4^cre^Chrna7^fl/fl^* mice for 5 days. (**B**) Expression of TSLPR, IL-17RA, and ST2 in lung Mks was tested by flow cytometry. (**C**) Determination of total cells and eosinophils in BALF by flow cytometry. (**D**) Determination of ILC2s in lung tissue by flow cytometry. (**E**) Representative H&E staining. Scale bar: 200 μm. (**F**) Severity score of lung tissue. Data are representative of at least 3 independent experiments and are presented as mean ± SD (*n* = 3–5). *P < 0.05; **P < 0.01; ***P < 0.001; ****P < 0.0001 by 1-way ANOVA with Tukey’s post hoc analysis (**C**, **D**, and **F**). NS, not significant.

Aiming to confirm the role of α7 nAChR on Mks in allergic airway inflammation, we generated *Pf4^cre^Chrna7^fl/fl^* mice to conditionally delete α7 nAChR on Mks. We then challenged *Chrna7^fl/fl^* mice and *Pf4^cre^Chrna7^fl/fl^* mice with PBS or papain for 5 days and assessed airway inflammation (Figure 3A). We also confirmed that platelets did not express α7 nAChR (Supplemental Figure 2A). Our experiments revealed that conditional deletion of α7 nAChR on Mks alleviated papain-induced allergic airway inflammation, including the decreased percentage and numbers of eosinophils in BALF (Figure 3B), decreased percentage and numbers of ILC2s (Figure 3C), alleviated airway inflammation (Figure 3, D and E), and reduced relative mRNA expression of *Il33*, *Il5*, and *Il13* in lung tissue (Figure 3F). Given that α7 nAChR^+^ Mks exhibited higher expression of ST2, we also tested the expression of ST2 on Mks after conditional knockout of α7 nAChR. We found that with the knockout of α7 nAChR, the expression of ST2 on Mks was correspondingly downregulated (Figure 3G). To exclude the effects of Pf4-cre activity on other immune cell subsets, we also quantified lymphocytes and other myeloid cells in lung tissue from both control and experimental groups. The numbers of CD4^+^ T cells, CD8^+^ T cells, DCs, monocytes, and neutrophils, as well as the proportion of Mks (which could not be accurately counted due to the FSC-high gating strategy) in lung tissue were not affected by Pf4-cre activity in either the control or inflammation model groups (Supplemental Figure 2B). In addition, the deletion of *Chrna7* on Mks did not affect the number of platelets, leukocytes, erythrocytes, hemoglobin, mean platelet volume, and mean corpuscular hemoglobin concentration in peripheral blood (Supplemental Figure 2C). In order to verify our findings, we challenged *Chrna7^fl/fl^* mice and *Pf4^cre^Chrna7^fl/fl^* mice with HDM extracts for 14 days or IL-33 for 3 days to induce other types of allergic airway inflammation (Figure 4A and Figure 5A). We found less inflammatory cell infiltration (Figure 4, B and C), lower percentages of Th2 cells in mediastinal lymph nodes (MLNs), and alleviated lung injury in *Pf4^cre^Chrna7^fl/fl^* mice compared with *Chrna7^fl/fl^* mice (Figure 4, D–G). However, knockout of α7 nAChR on Mks did not affect Th17 cells in MLNs (Figure 4E). These data suggest that α7 nAChR on Mks play an important role in the pathogenesis of HDM-induced type 2 airway inflammation in vivo. We have found that lung Mks express ST2 (Figure 2F), which indicated that Mks were regulated by IL-33/ST2 signaling, so we tested protein expression of the alarmin receptor ST2 on lung Mks. As shown in Figure 5B, we found ST2 expression was significantly higher than that of the thymic stromal lymphopoietin (TSLP) receptor (TSLPR) and IL-17RA (IL-25 receptor) on lung Mks by flow cytometry. Similar results were observed in mouse models of allergic airway inflammation induced by IL-33 (Figure 5, C–F); knockout of α7 nAChR on Mks alleviated IL-33–induced allergic airway inflammation.

**Figure 6:**
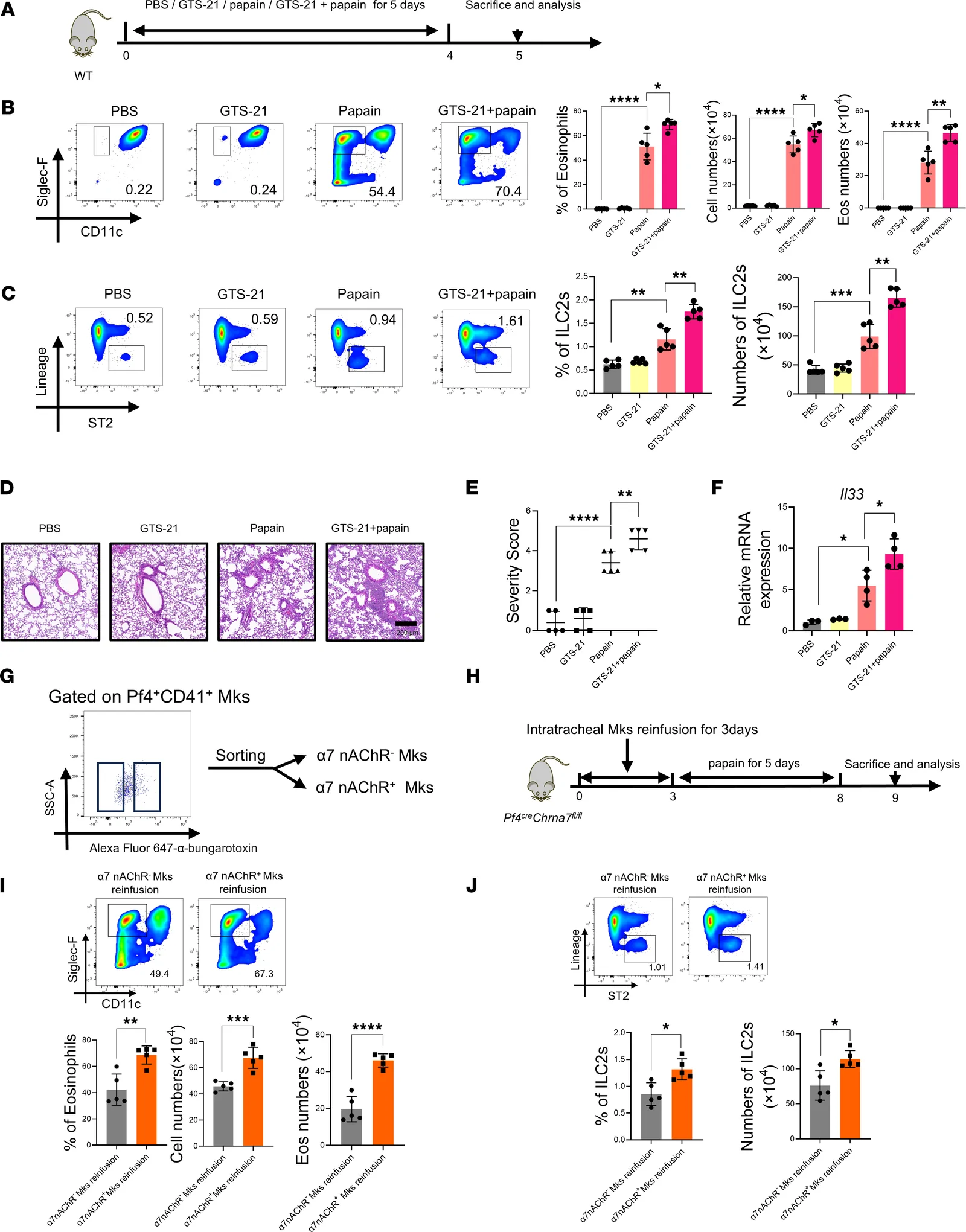
α7 nAChR agonist GTS-21 aggravates papain-induced airway inflammation. (**A**) Schematic diagram of GTS-21 intervention in papain-induced allergic airway inflammation model. (**B**) Determination of total cells and eosinophils in BALF by flow cytometry. (**C**) Determination of ILC2s in lung tissue by flow cytometry. (**D**) Representative H&E staining and severity score of lung histology. Scale bar: 200 μm. (**E**) Severity score of lung histology. (**F**) The relative mRNA expression of *Il33* in lung tissue homogenate tested by RT-qPCR. (**G**) The flow cytometric gating strategy for sorting α7 nAChR^–^ Mks and α7 nAChR^+^ Mks. (**H**) α7 nAChR^–^ and α7 nAChR^+^ Mks were intratracheally delivered to *Pf4^cre^Chrna7^fl/fl^* mice (1 × 10^6^ Mks per mouse) in the papain-induced allergic airway inflammation model. (**I**) Determination of total cells, eosinophils, and neutrophils in BALF by flow cytometry. (**J**) Determination of ILC2s in lung tissue by flow cytometry. Data are representative of at least 3 independent experiments and are presented as mean ± SD (*n* = 3–5). *P < 0.05; **P < 0.01; ***P < 0.001; ****P < 0.0001 by 1-way ANOVA with Tukey’s post hoc analysis (**B**, **C**, **E**, and **F**) or 2-sided *t* test (**I** and **J**). NS, not significant.

To further demonstrate that activation of α7 nAChR on lung Mks aggravates allergic airway inflammation, we treated WT mice with PBS, GTS-21, papain, or GTS-21 plus papain for 5 days (Figure 6A). As expected, we noticed that activation of α7 nAChR amplified papain-induced allergic inflammation. GTS-21 increased the percentage and numbers of eosinophils in BALF and ILC2s in lung (Figure 6, B–E) and promoted the *Il33* expression of lung tissue in papain-challenged groups (Figure 6F). We also tested the number of peripheral blood platelets in this experiment, and no differences were observed in the 4 groups (Supplemental Figure 3A). As shown in Supplemental Figure 3, B and C, α7 nAChR on Mks had no effect on relative gene expression of *Tslp* and *Il25* in lung tissue. Given that knockout of α7 nAChR on Mks alleviates papain-induced airway inflammation and GTS-21 aggravates airway inflammation, we next treated *Pf4^cre^Chrna7^fl/fl^* mice with PBS plus papain or GTS-21 plus papain for 5 days (Supplemental Figure 4A). Interestingly, we found that GTS-21 failed to amplify airway inflammation in *Pf4^cre^Chrna7^fl/fl^* mice (Supplemental Figure 4B). These results demonstrated that the proinflammatory effect of GTS-21 was dependent on Mks. To verify the proinflammatory role of α7 nAChR^+^ Mks, we sorted α7 nAChR^–^ and α7 nAChR^+^ lung Mks and transfused them back into *Pf4^cre^Chrna7^fl/fl^* mice before papain challenge for 3 days (Figure 6, G and H). We found α7 nAChR^+^ Mk reinfusion aggravated papain-induced airway inflammation compared with α7 nAChR^–^ Mk reinfusion, characterized by more eosinophil and ILC2 infiltration (Figure 6, I and J). Collectively, these results indicate that activation of α7 nAChR on Mks amplifies allergic airway inflammation.

### Activation of α7 nAChR promotes Mk IL-33 expression and release through p38 MAPK signaling pathway.

**Figure 7:**
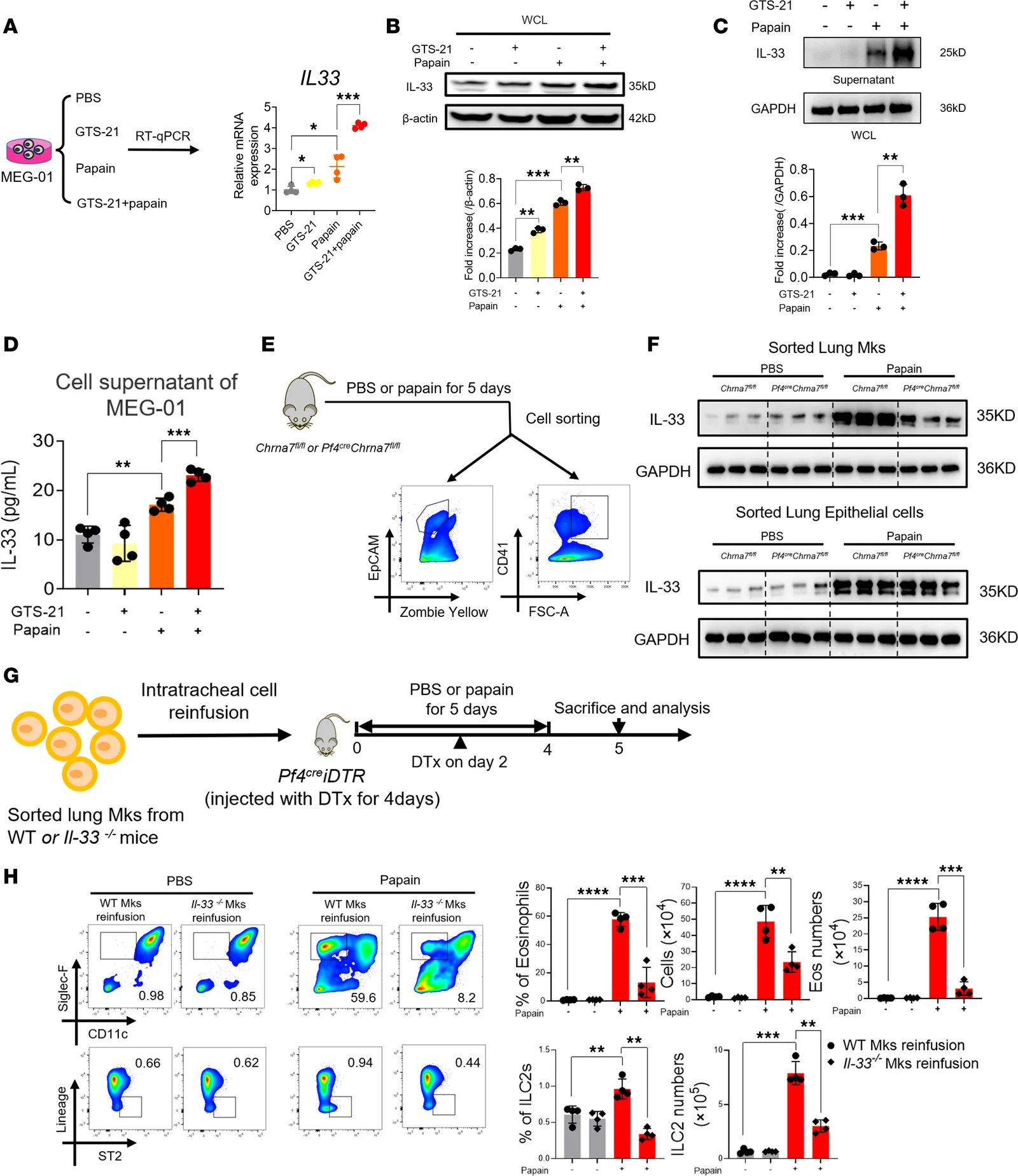
Activation of α7 nAChR on lung Mks aggravates airway inflammation via IL-33 release. (**A**) The relative *IL33* mRNA expression in MEG-01 cells cultured with GTS-21 and papain was tested by RT-qPCR and Western blotting. (**B**) The expression of IL-33 (35 kDa) protein in MEG-01 was tested by Western blotting. WCL, whole-cell lysate. (**C**) The IL-33 (25 kDa) protein in supernatant of MEG-01 was tested by Western blotting. (**D**) The release of IL-33 in supernatant of MEG-01 was tested by ELISA. (**E**) The flow cytometric gating strategy for sorting lung epithelial cells and Mks. (**F**) The expression of IL-33 (35 kDa) protein in lung epithelial cells and Mks was tested by Western blotting. (**G**) Schematic diagram of intratracheal Mk reinfusion into *Pf4^cre^iDTR* mice. (**H**) Determination of eosinophils in BALF and ILC2s in lung tissue by flow cytometry. Data are representative of at least 3 independent experiments and are presented as mean ± SD (*n* = 3–5). *P < 0.05; **P < 0.01; ***P < 0.001; ****P < 0.0001 by 1-way ANOVA with Tukey’s post hoc analysis (**A**–**D** and **H**). NS, not significant.

To determine how α7 nAChR on Mks affects airway inflammation, we cultured the MEG-01 cell line in vitro with PBS, GTS-21, papain, or GTS-21 plus papain. Considering the decreased mRNA expression of *Il33* in *Pf4^cre^Chrna7^fl/fl^* mice, we hypothesized that α7 nAChR had a regulatory effect on IL-33 expression in Mks. As we expected, Mks cultured with GTS-21 upregulated relative mRNA and protein expression of IL-33 compared with Mks cultured with papain alone (Figure 7, A and B). Through Western blot and enzyme-linked immunosorbent assay (ELISA) detection of secretory proteins in the supernatant of the culture system, we also found that activation of α7 nAChR promoted IL-33 release from Mks (Figure 7, C and D). IL-33 is an epithelial cell–derived cytokine that is predominantly expressed in lung AECs during allergic airway inflammation (31). To eliminate the impact of AEC-derived IL-33 on the results, we sorted AEC adhesion molecule–positive (EpCAM^+^) AECs and CD41^+^, FSC-high megakaryocytes from murine lung tissues to detect IL-33 expression levels (Figure 7E). We found that compared with *Chrna7^fl/fl^* mice, IL-33 expression in lung epithelial cells of *Pf4^cre^Chrna7^fl/fl^* mice remained unchanged, whereas IL-33 expression in lung Mks was significantly reduced during airway inflammation (Figure 7F). To further demonstrate the importance of IL-33 in Mks during airway inflammation, we sorted lung Mks from *Il33^–/–^* or WT mice and administered them via intratracheal instillation into *Pf4^cre^iDTR* mice (Figure 7G), whose Mks had been depleted by DT (1 × 10^6^ cells per mouse). We then reestablished the inflammation model to assess airway inflammation. Compared with WT controls, inflammation levels were significantly milder in mice receiving lung Mks from *Il33^–/–^* mice (Figure 7H). Furthermore, GTS-21 did not upregulate *Il33* expression in BEAS-2B, an AEC cell line (Supplemental Figure 5). Considering the ability of Mks to process antigens such as OVA, we used DQ-OVA (Thermo Fisher D120553) to test whether activation of α7 nAChR affects OVA processing. We incubated MEG-01 with or without GTS-21 (50 μM) for 2 hours, then treated the cells with DQ-OVA (200 μg/mL) and 30 minutes later determined fluorescence by flow cytometry. Consistent with previous reports, Mks significantly internalized DQ-OVA. However, the GTS-21 had no effect on this process (Supplemental Figure 6A). We also tested Mk exosomes after GTS-21 treatment. As shown, activation of α7 nAChR does not affect the release of exosomes by Mks (Supplemental Figure 6B).

**Figure 8:**
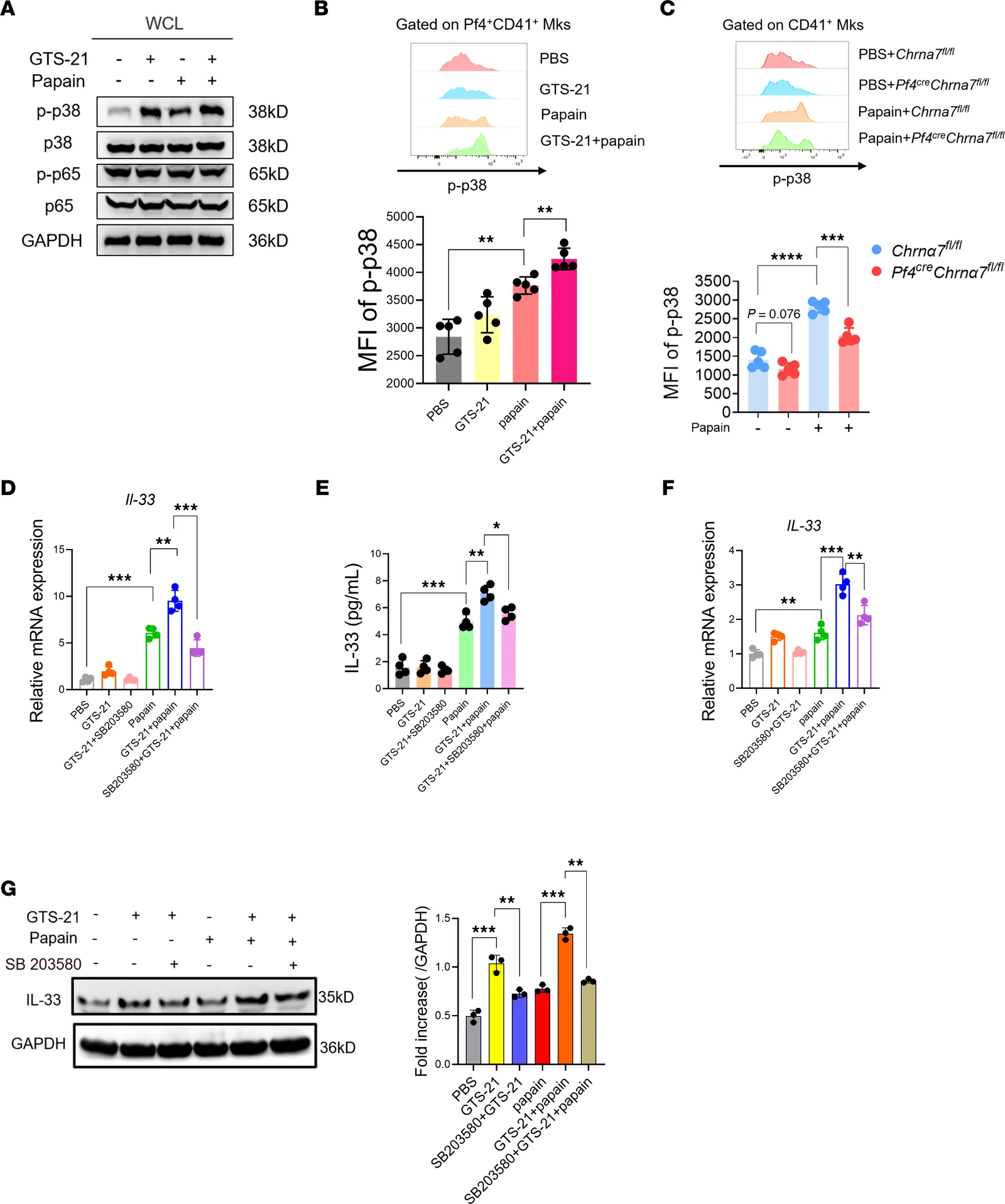
Activation of α7 nAChR promotes Mk IL-33 expression and release through p38 MAPK signaling pathway. (**A**) The phosphorylation of p38 and p65 was assessed by Western blotting. (**B**) The phosphorylation of p38 in lung Mks was evaluated in a papain-induced allergic airway inflammation model with GTS-21 intervention by flow cytometry. (**C**) The phosphorylation of p38 in lung Mks was tested in *Chrna7^fl/fl^* and *Pf4^cre^Chrna7^fl/fl^* mice with papain challenge. (**D**) The relative mRNA expression of *Il33* in lung Mks cultured with GTS-21, papain, and SB203580 was tested by RT-qPCR. (**E**) The release of IL-33 in supernatant of lung Mks was assessed by ELISA. (**F**) The relative mRNA expression of *IL33* in MEG-01 cultured with GTS-21, papain, and SB203580 was evaluated by RT-qPCR. (**G**) The expression of IL-33 (35 kDa) protein in MEG-01 cultured with GTS-21, papain, and SB203580 was assessed by Western blotting. Data are representative of at least 3 independent experiments and are presented as mean ± SD (*n* = 3–5). *P < 0.05; **P < 0.01; ***P < 0.001; ****P < 0.0001 by 1-way ANOVA with Tukey’s post hoc analysis (**B**–**G**). NS, not significant.

Next, we investigated which signaling pathway mediates the proinflammatory effect of α7 nAChR on Mks. Via Western blot, we found that GTS-21 promoted phosphorylation of p38 MAPK in Mks. For comparison, we also examined the NF-κB signaling pathway and found that GTS-21 did not affect the phosphorylation level of p65 (Figure 8A). To verify our findings, we tested the phosphorylation of p38 MAPK in lung Mks. Consistent with the cell line results, GTS-21 promoted phosphorylation of p38 MAPK in Mks, while knockout of α7 nAChR inhibited this process (Figure 8, B and C). Then we utilized a selective p38 MAPK inhibitor (SB203580) to suppress the phosphorylation of p38 MAPK in sorted lung Mks and MEG-01. We found that SB203580 treatment restrained the increased gene expression of *Il33* and IL-33 protein release in lung Mks caused by GTS-21 (Figure 8, D and E). These findings were further confirmed in MEG-01 cells (Figure 8, F and G). In summary, these results suggest that overexpression of IL-33 caused by α7 nAChR activation on Mks is dependent on phosphorylation of p38 MAPK.

## Discussion

It has been reported that there are a large number of Mks residing in lung tissues, and it has been found that they can produce a large number of platelets and release them into the blood. They can even be used as a backup “hematopoietic pool” to supplement the missing Mks in BM and participate in BM hematopoiesis (14). A study has compared lung Mks with BM Mks and found that lung Mks express more costimulatory molecules and have functions similar to DCs, such as OVA antigen processing (15). This suggests that Mks have potential important functions in autoimmune diseases, such as allergic airway inflammation. There have been corresponding reports on Mks in other diseases. For example, a large number of abnormal Mks were found in the lung tissues of patients with COVID-19 pneumonia, and patients with COVID-19 pneumonia showed a large extent of platelet consumption (35). However, there are still few studies on the immunoregulatory role of Mks in allergic airway diseases.

In previous study, platelets were found to have a proinflammatory effect on papain-induced allergic inflammation (17). Here in our study, we discovered that Mks were increased in the lung tissues of asthmatic model mice and exacerbate allergic airway inflammation, which were independent of platelets. Neuro-immune crosstalk has been found to play an important role in multiple diseases (36). Some studies revealed that neurotransmitters such as acetylcholine (37), calcitonin gene–related peptides (11, 38, 39), and dopamine (40) have regulatory effect on immune cells in allergic airway inflammation. In our previous studies, we found that the vagus nerve/α7 nAChR axis had the potential to regulate the process of lung injury (27), virus replication (24, 41, 42), and cell migration (26). To investigate whether Mks express α7 nAChR, we used α-bungarotoxin to label α7 nAChR on lung cells and BM cells. Our results demonstrated that α7 nAChR was expressed in both lung and BM Mks. Notably, the expression level was substantially higher in lung Mks than in BM Mks, suggesting heterogeneity between the 2 cell populations. At the same time, compared with the α7 nAChR^–^ Mks, the α7 nAChR^+^ Mks express more ST2 protein, indicating that IL-33 has a more pronounced regulatory effect on the α7 nAChR^+^ Mks. After binding to cell-surface ST2, IL-33 activates NF-κB, JUNK, ERK, and other signaling pathways through the Myd88 pathway, which in turn stimulates downstream immune responses (43). In allergic asthma, IL-33 also plays an important proinflammatory role as a key alarmin molecule, which can promote the maturation, proliferation, and enhancement of the functions of ILC2s, Th2 cells, and other cells (44). This suggests that the α7 nAChR^+^ Mks may play a role in responding to IL-33 in allergic asthma.

Our current study shows that both α7 nAChR and ST2 are expressed on Mks and exhibit significant proinflammatory effects. In our unpublished bulk RNA-seq data from MEG-01 cells stimulated with LPS and GTS-21, we found that α7 nAChR activation by GTS-21 increased expression of *MAPK15* (which encodes p38 MAPK), *NFKB2*, and *IL1RL1* (ST2) under both baseline and LPS-challenged conditions; *IL33* expression also showed an increasing trend, whereas JAK/STAT3 signaling was unaffected. Although MEG-01 is a BM-derived cell line and differs from lung Mks, the underlying mechanism remains consistent; α7 nAChR activation boosts p38 MAPK and IL-33/ST2 signaling.

Papain is a protease from papaya used to induce an asthma-like allergic response characterized by airway hyperresponsiveness, mucus production, and immune cell infiltration. IL-33 is an alarmin cytokine involved in type 2 inflammatory responses (e.g., asthma), promoting the activation of Th2 cells, eosinophils, and mast cells. In both models, Mk-specific knockout of α7 nAChR resulted in lower levels of airway inflammation, decreased immune cell infiltration, and reduced proinflammatory cytokine production, suggesting that α7 nAChR activation in lung Mks plays a role in driving inflammation in allergic airway diseases. Modulating this receptor could provide a means to control inflammation without affecting the broader cholinergic system that regulates other bodily functions. This approach may offer a more targeted strategy to treat asthma and similar conditions by specifically targeting the cells that exacerbate inflammation, like Mks, rather than indiscriminately affecting nicotinic receptors in other tissues.

While α7 nAChR activation typically suppresses inflammation via the cholinergic antiinflammatory pathway in macrophages (e.g., via upregulating JAK2/STAT3 pathways) (45, 46), its activation in lung Mks paradoxically exacerbates allergic airway inflammation. This finding challenges the prevailing view of α7 nAChR as a universal antiinflammatory target, emphasizing the need for cell-specific therapeutic strategies in asthma or chronic airway diseases. This study also reveals the dual roles of α7 nAChR depending on cellular context (47), enriching our understanding of receptor biology and inflammation regulation‌.

The p38 MAPK pathway is a critical signaling cascade involved in the regulation of inflammation and immune responses. In our previous studies, we found that the interaction between α7 nAChR activation, p38 MAPK signaling, and viral replication is crucial for understanding the pathogenesis of among influenza, HIV-1, and ZIKV infections. In influenza, α7 nAChR activation promotes viral replication by inhibiting p38 MAPK phosphorylation, which facilitates viral ribonucleoprotein export (41). In HIV-1, α7 nAChR activation enhances HIV-1 transcription via the ROS/p-p38MAPK/LMNB1/NFATC4 signaling pathway (24). The activation of α7 nAChR represents a promising approach to limit ZIKV infection through a combination of enhancing autophagy and promoting ferroptosis (42). In this study, by using conditional knockout mice and MEG-01, we found that activation of α7 nAChR on Mks accelerated allergic airway inflammation by increased IL-33 expression via the p38 MAPK signaling pathway. This dual role of α7 nAChR activation underscores the complexity of its effects across different viral pathogens and the importance of targeting this receptor with careful consideration in developing therapeutic interventions.

In conclusion, the Mk-specific knockout of α7 nAChR in papain and IL-33 challenge models provides crucial evidence that α7 nAChR in lung Mks plays a significant role in worsening allergic airway inflammation. This study highlights α7 nAChR as a critical player in immune modulation and points to p38 MAPK activation as a key pathway driving inflammation. Importantly, it suggests that targeting α7 nAChR in lung Mks could offer a new therapeutic approach to managing asthma and potentially other chronic inflammatory diseases, by reducing inflammation without affecting other cholinergic functions in the body.

## Methods

### Sex as a biological variable.

Both male and female mice were included in this study. Sex was considered as a biological variable in the study design, data analysis, and reporting.

### Animals.

WT C57BL/6J mice were purchased from the Shanghai Laboratory Animal Center. *Pf4^Cre^* mice on a C57BL/6J background were provided by Yi Wu (The Cyrus Tang Hematology Center, Collaborative Innovation Center of Hematology, Soochow University, Suzhou, China). *Chrna7^fl/fl^* mice were generated by the Transgenic Mouse Facility led by Yan Zhang in the Institute Pasteur of Shanghai, China. B6.Cg-*Gt(ROSA)26Sort^m9(CAG-tdTomato)Hze^*/J (Ai9) mice were provided by Zilong Qiu (Institute of Neuroscience, State Key Laboratory of Neuroscience, Chinese Academy of Sciences, Shanghai, China). C57BL/6-*Gt(ROSA)26Sor^tm1(HBEGF)Awai^*/J (iDTR) mice were provided by Rui Zeng (Tongji Hospital, Tongji Medical College, Huazhong University of Science and Technology, Hubei, China) and *Il33^–/–^* mice were provided by Guochao Shi (Department of Pediatrics, Ruijin Hospital, Shanghai Jiao Tong University School of Medicine, Shanghai, China). *Pf4^cre^* mice were used to cross with Rosa26-tdTomato mice to generate *Pf4^cre^tdTomato* mice to label Mks. *Pf4^cre^* mice were used to cross with Rosa26-*iDTR* mice for conditional ablating Mks by DT injection. *Pf4^cre^* mice were used to cross with *Chrna7^fl/fl^* mice for conditional knockout of *Chrna7* in Mks.

### Depletion of Mks and platelet transfer in Pf4^cre^iDTR mice.

To deplete Mks in vivo, female *Pf4^cre^iDTR* mice (6–8 weeks old) were injected with 200 ng DT (Sigma-Aldrich) in 100 μL PBS on days 0, 1, 2, 3, 4, and 7. Flow cytometry was used to test the efficiency of depletion. Donor platelets were obtained by mouse peripheral blood platelet isolation kit (Solarbio) from WT C57BL/6J mice. Each recipient mouse received 4 × 10^8^ donor platelets per transfer through tail vein injection on days 2, 5, and 7.

### Allergic airway inflammation models.

To induce OVA-specific asthma, female mice were sensitized by intraperitoneal (i.p.) injection of 100 μg OVA (Sigma-Aldrich) on days 0 and 14, and subsequently challenged intranasally with 100 μg OVA on days 23, 24, and 25. To establish papain-induced allergic airway inflammation, female mice (6–8 weeks old) were anesthetized by isoflurane inhalation and then administered 10 μg papain (Sigma-Aldrich) in 40 μL PBS via the intranasal route for 5 consecutive days. For IL-33–induced allergic airway inflammation, female mice (6–8 weeks old) were anesthetized by isoflurane inhalation and sensitized intranasally with 200 ng recombinant mouse IL-33 (BioLegend) in 40 μL PBS for 3 consecutive days. Samples of these 3 airway inflammation models were collected and analyzed 24 hours after the last stimulation. For HDM-induced type 2 inflammatory immune responses, female mice (6–8 weeks old) were challenged intranasally with 100 μg of HDM (Greer Laboratory) extracts on day 0 and 10 μg of HDM extracts from day 7 to day 11. Mice were anesthetized and analyzed on day 14.

### Preparation of single-cell samples.

Mice were sacrificed humanely and BALF of the whole lung was collected by syringes in 1 mL PBS. BALF was centrifuged at 300*g* for 10 minutes at 4°C. Cells were resuspended in FACS buffer (2% FBS, 1% penicillin, 1% streptomycin, and 0.5% EDTA) at 4°C. Lung tissue was incubated in Hanks’ balanced salt solution (HBSS) containing 1 mg/mL collagenase I and Dispase II (Thermo Fisher 17100017 and 17105041) at 37°C for 40 minutes. The suspension was thoroughly mixed and passed through a 70 μm cell strainer. After that, the cell pellet was incubated in RBC lysis buffer (Beyotime) for 3 minutes to eliminate RBCs in the lung. Cells were centrifuged at 300*g* for 7 minutes at 4°C and resuspended in FACS buffer for further analysis.

### Flow cytometry.

Anti–mouse CD16/CD32 antibodies were used to block Fc receptors and a Zombie Yellow Fixable Viability Kit (Biolegend 423104) was used to exclude dead cells. Then, BALF and lung cells were incubated with antibodies for 30 minutes at 4°C in dark room. The antibodies used were as follows: APC anti-mouse CD41, MWReg30; FITC anti-mouse CD41, MWReg30; PE anti-mouse Siglec-F, S17007L; APC anti-mouse CD11c, N418; FITC anti-mouse NK1.1, S17016D; CD3e, 500A2; CD11b, M1/70; Gr-1, RB6-8C5; Ter119, TER-119; TCRβ, H57-597; TCR γ/δ, UC7-13D5; CD4,GK1.5; CD19,6D5, CD8a, S18018E; CD3, 17A2; CD45R, RA3-6B2; PE anti-mouse CD127, S18006K; PE-Cy7 anti-mouse CD90.2, 30-H12; Brilliant Violet 421 anti-mouse ST2, DIH9; Brilliant Violet 711 anti-mouse CD11c, N418; PE-Cy7 anti-mouse MHC-II, M1/42; APC anti-mouse TSLPR,22H9 (BioLegend); APC-Cy7 anti-mouse CD45, 30-F11 (BD Biosciences); APC anti-mouse ST2, RMST2-2; APC anti-mouse IL-17RA, PAJ-17R (Invitrogen); and Alexa Fluor 647–conjugated a-bungarotoxin B35450 (Biotium). After incubation, cells were washed twice and resuspended in FACS buffer. The samples were analyzed on a Fortessa (BD Biosciences) and the results were analyzed by FlowJo (Tree Star). For cell sorting, the samples were processed on a FACSAria Fusion (BD Biosciences) and the cells were collected for further experiments.

### Vagus nerve electrical signal measurement.

The vagus nerve recordings were performed as described previously (48). Briefly, mice were anesthetized with 3% isoflurane in 100% air at a flow rate of 0.9 L/min for 3 minutes. Then, mice were maintained at 1.5% isoflurane and placed on a heating pad to maintain body temperature at approximately 37°C. To isolate the cervical vagus nerve, the neck area was cleaned with iodine, and a midline cervical incision was made from the level of larynx to sternum. Submaxillary salivary glands were separated to expose the trachea. The right cervical vagus nerve was delicately separated from the artery and desheathed by gently removing the thin connective tissue surrounding the nerve under magnification. Then, the vagus nerve was placed on 2 hook electrodes with mineral oil to insulate the nerve and surrounding tissue, and then the ground electrode was inserted to the mouse’s tail. A PowerLab 4/26 data acquisition system (ADInstruments) was used to record vagus nerve signals. Recordings were sampled at 40 kHz, 120 Hz filter, and 100 gain to ensure stability of the signal during the recording period of 10 minutes. The analytical process has been previously described (48). In brief, signals in mat format were exported from LabChart (ADInstruments) and analyzed in MATLAB (MathWorks). Signals were decomposed into numerous single spikes and the action potentials were detected with a threshold determined by smallest of constant false-alarm rate (SO CFAR) filter. The t-SNE method was used to perform dimensionality reduction of all spike data and the DBSCAN method (48) was used to perform cluster analysis of all spikes. All spikes were then clustered into different groups based on the waveforms. All significative waveforms were counted and their distribution over time graphed and analyzed.

### Real-time quantitative PCR.

Total RNA of cells or lung homogenates was extracted by using TRIzol reagent (Invitrogen). RNA was reverse transcribed into cDNA by synthesis kits (Yeasen). Then, real-time quantitative PCR (RT-qPCR) was performed by SYBR Green master mix (Yeasen) in a RT-qPCR system. The primers we used are listed in Supplemental Table 1. The relative mRNA expression was analyzed using Excel (Microsoft).

### In vitro cell culture and treatment.

The human Mk cell line MEG-01 (ATCC) was cultured in RPMI-1640 medium containing 10% FBS and 1% penicillin/streptomycin. The human AEC line BEAS-2B (ATCC) was cultured in DMEM containing 10% FBS and 1% penicillin/streptomycin. Lung Mks were sorted by FACSAria Fusion and cultured in RPMI-1640 medium containing 15% FBS and 1% penicillin/streptomycin. Cells were treated with 50 μM GTS-21 (MedChemExpress), 50 μg/mL papain (Merck), and 20 μM SB203580 (MedChemExpress) for stimulation. Cells and culture medium were collected for further study.

### Western blot.

Cells were lysed in cell lysis buffer for Western and IP (Beyotime) with protease and phosphatase inhibitors at 4°C for 20 minutes. Then, supernatant was obtained after centrifugation at 10,000*g* for 10 minutes and boiled in loading buffer (Epizyme). The protein expression was analyzed by SDS-PAGE. The antibodies applied were as follows: GAPDH, 1E6D9 and EPR16891; IL-33, EPR17831 and 2A9C7 (Proteintech and Abcam); p38, sc-81621; p-p38, sc-7973; p65, sc-109; p-p65, sc-33020 (Santa Cruz Biotechnology); β-actin, 10C9B3 (Servicebio); ALIX, EPR23653-32; TSG101, EPR7130; CD81, M38 (Abcam).

### Concentration of cell culture medium.

Cell culture medium was collected in 15 mL centrifuge tubes and placed in a freeze-drying machine for 24 hours after being rapidly frozen in liquid nitrogen. Then, 100 μL of double-distilled water was added for resuspension and used for cytokine detection.

### ELISA.

Analysis of IL-33 in supernatant was performed by ELISA kit (Multi Sciences) according to the manufacturer’s directions after concentration of culture medium.

### Lung Mk intratracheal reinfusion.

Single-cell samples from lung tissue were prepared from *Pf4^cre^tdTomato* mice as described above. Then,we sorted CD41^+^tdTomato^+^α-bungarotoxin^+^ cells as α7 nAChR^+^ Mks and CD41^+^tdTomato^+^α-bungarotoxin^–^ cells as α7 nAChR^–^ Mks by flow cytometry. α7 nAChR^–^ and α7 nAChR^+^ Mks were intratracheally delivered to *Pf4^cre^Chrna7^fl/fl^* mice (1 × 10^6^ Mks per mouse) in the papain-induced allergic airway inflammation model before papain challenge for 5 days. A similar procedure was used to isolate lung Mks from WT and *Il33*^–/–^ mice for intratracheal reinfusion.

### Statistics.

Data are represented as the mean ± SD and statistical analyses were performed using GraphPad Prism v8.0. For all experiments, the difference between groups was calculated with a Student’s *t* test or ANOVA. A *P* value of less than 0.05 was considered significant.

### Study approval.

All animal experiments were conducted in accordance with the Institutional Animal Care and Use Committee guidelines of Shanghai Institute of Immunity and Infection, Chinese Academy of Sciences (Animal Ethics Review Number: A2024031).

## Author contributions

HW and RT performed most of the experiments and data analysis. HW and RT shared co–first authorship; the order of co–first authors was determined based on their contribution to the study. HW, XS, ZX, and JFX designed the experiments and wrote the manuscript. SX assisted with the electrical signal acquisition experiments. YZ assisted with mouse breeding, sample collection, and provided technical guidance. JFX, ZX, and XS supervised the study, provided scientific insight, and reviewed and edited the manuscript.

## Conflict of interest

The authors have declared that no conflict of interest exists.

## Funding support

- National Science Foundation of China programs 82495200/02, 82470024, and 82170035.
- Innovative Research Team of High-level Local Universities in Shanghai grant SHSMU-ZDCX20210602.
- Science and Technology Commission of Shanghai Municipality grant 20DZ2261200.

## Supplementary Material

Supplemental data

Supplemental data set 1

Unedited blot and gel images

Supporting data values

## Version 1 09/08/2026

Electronic publication

## References

[1] Miller RL (2021). Advances in asthma: new understandings of asthma’s natural history, risk factors, underlying mechanisms, and clinical management. J Allergy Clin Immunol.

[2] Hammad H, Lambrecht BN (2021). The basic immunology of asthma. Cell.

[3] Froidure A (2016). Dendritic cells revisited in human allergic rhinitis and asthma. Allergy.

[4] LeSuer WE (2023). Eosinophils promote effector functions of lung group 2 innate lymphoid cells in allergic airway inflammation in mice. J Allergy Clin Immunol.

[5] Suzuki Y (2017). Airway basophils are increased and activated in eosinophilic asthma. Allergy.

[6] Curren B (2023). IL-33-induced neutrophilic inflammation and NETosis underlie rhinovirus-triggered exacerbations of asthma. Mucosal Immunol.

[7] Pejler G (2019). The emerging role of mast cell proteases in asthma. Eur Respir J.

[8] Lechner A (2022). Macrophages acquire a TNF-dependent inflammatory memory in allergic asthma. J Allergy Clin Immunol.

[9] Lambrecht BN, Hammad H (2015). The immunology of asthma. Nat Immunol.

[10] Komlosi ZI (2022). Cellular and molecular mechanisms of allergic asthma. Mol Aspects Med.

[11] Sui P (2018). Pulmonary neuroendocrine cells amplify allergic asthma responses. Science.

[12] Stone AP (2022). The bone marrow niche from the inside out: how megakaryocytes are shaped by and shape hematopoiesis. Blood.

[13] Wang J (2022). CXCR4^high^ megakaryocytes regulate host-defense immunity against bacterial pathogens. Elife.

[14] Lefrancais E (2017). The lung is a site of platelet biogenesis and a reservoir for haematopoietic progenitors. Nature.

[15] Pariser DN (2021). Lung megakaryocytes are immune modulatory cells. J Clin Invest.

[16] Petzold T (2022). Neutrophil “plucking” on megakaryocytes drives platelet production and boosts cardiovascular disease. Immunity.

[17] Takeda T (2016). Platelets constitutively express IL-33 protein and modulate eosinophilic airway inflammation. J Allergy Clin Immunol.

[18] Noviello CM (2021). Structure and gating mechanism of the α7 nicotinic acetylcholine receptor. Cell.

[19] Wang H (2003). Nicotinic acetylcholine receptor alpha7 subunit is an essential regulator of inflammation. Nature.

[20] Kwok HH (2021). Nicotinic acetylcholine receptor subunit α7 mediates cigarette smoke-induced PD-L1 expression in human bronchial epithelial cells. Cancers (Basel).

[21] Keever KR (2024). Cholinergic signaling via the α7 nicotinic acetylcholine receptor regulates the migration of monocyte-derived macrophages during acute inflammation. J Neuroinflammation.

[22] Galle-Treger L (2016). Nicotinic acetylcholine receptor agonist attenuates ILC2-dependent airway hyperreactivity. Nat Commun.

[23] Yue-Chun L (2021). Vagus nerve plays a pivotal role in CD4^+^ T cell differentiation during CVB3-induced murine acute myocarditis. Virulence.

[24] Wen J (2022). Activation of α7 nicotinic acetylcholine receptor promotes HIV-1 transcription. Cell Insight.

[25] Kong W (2018). GTS-21 protected against LPS-induced sepsis myocardial injury in mice through α7nAChR. Inflammation.

[26] Zhao C (2017). Signals of vagal circuits engaging with AKT1 in α7 nAChR^+^CD11b^+^ cells lessen *E*. *coli* and LPS-induced acute inflammatory injury. Cell Discov.

[27] Chen X (2023). α7nAChR activation in AT2 cells promotes alveolar regeneration through WNT7B signaling in acute lung injury. JCI Insight.

[28] Hong H (2020). Role of IL-25, IL-33, and TSLP in triggering united airway diseases toward type 2 inflammation. Allergy.

[29] Afferni C (2018). The pleiotropic immunomodulatory functions of IL-33 and its implications in tumor immunity. Front Immunol.

[30] Cephus JY (2021). Estrogen receptor-α signaling increases allergen-induced IL-33 release and airway inflammation. Allergy.

[31] Chen W (2022). Allergen protease-activated stress granule assembly and gasdermin D fragmentation control interleukin-33 secretion. Nat Immunol.

[32] Middleton EA (2018). Amicus or adversary revisited: platelets in acute lung injury and acute respiratory distress syndrome. Am J Respir Cell Mol Biol.

[33] Zhou Y (2019). Megakaryocytes participate in the occurrence of bleomycin-induced pulmonary fibrosis. Cell Death Dis.

[34] Huang DY (2022). Megakaryocytes in pulmonary diseases. Life Sci.

[35] Fortmann SD (2023). Circulating SARS-CoV-2^+^ megakaryocytes are associated with severe viral infection in COVID-19. Blood Adv.

[36] Chu C (2020). Neuro-immune interactions in the tissues. Immunity.

[37] Roberts LB (2022). Differential regulation of allergic airway inflammation by acetylcholine. Front Immunol.

[38] Tamari M (2024). Sensory neurons promote immune homeostasis in the lung. Cell.

[39] Wallrapp A (2019). Calcitonin gene-related peptide negatively regulates alarmin-driven type 2 innate lymphoid cell responses. Immunity.

[40] Cao Y (2023). Dopamine inhibits group 2 innate lymphoid cell-driven allergic lung inflammation by dampening mitochondrial activity. Immunity.

[41] Zhao C (2024). Vagal-α7 nicotinic acetylcholine receptor signaling exacerbates influenza severity by promoting lung epithelial cell infection. J Med Virol.

[42] Zhao C (2024). Activation of nicotinic acetylcholine receptor α7 subunit limits Zika viral infection via promoting autophagy and ferroptosis. Mol Ther.

[43] Xu L (2018). The IL-33-ST2-MyD88 axis promotes regulatory T cell proliferation in the murine liver. Eur J Immunol.

[44] Toki S (2020). TSLP and IL-33 reciprocally promote each other’s lung protein expression and ILC2 receptor expression to enhance innate type-2 airway inflammation. Allergy.

[45] Tracey KJ (2002). The inflammatory reflex. Nature.

[46] Yang X (2014). A novel regulator of lung inflammation and immunity: pulmonary parasympathetic inflammatory reflex. QJM.

[47] Chen J (2024). Neuroimmune recognition and regulation in the respiratory system. Eur Respir Rev.

[48] Zanos TP (2017). Identification of cytokine-specific sensory neural signals by decoding murine vagus nerve activity. Proc Natl Acad Sci U S A.

