# Supplementary material for: Activation of lung megakaryocyte α7 nAChR exacerbates allergic airway inflammation via IL-33/p38 MAPK signaling
Source: JCI Insight. 2026 Sep 8;11(17):e202201. doi: 10.1172/jci.insight.202201 (PMC13564082; doi:10.1172/jci.insight.202201)
Supplement: Supplementary file 1 [file jciinsight-11-202201-s274.pdf]

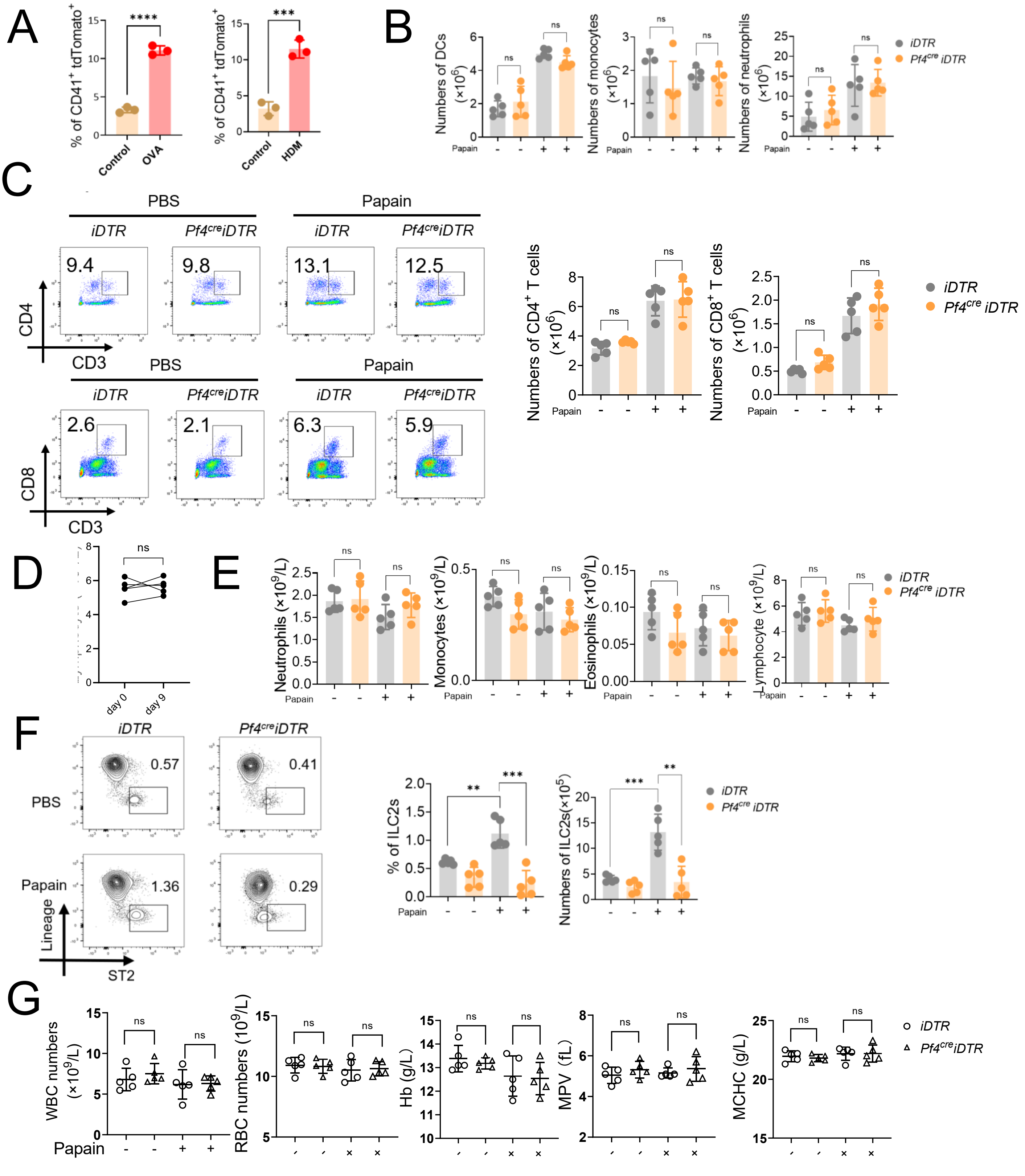

Figure S1. Influence on other immune cells in lung tissue and peripheral blood after depletion of Mks. A. The percentage of Mks were tested by flow cytometry in OVA and HDM group with respective control groups. B. The numbers of DCs, monocytes and neutrophils in lung tissue were determined by flow cytometry. C. The percentage and numbers of $CD4^+$ or $CD8^+$ T cells in lung tissue were determined by flow cytometry. D. The numbers of blood lymphocytes of *Pf4^cre iDTR* mice on day 0 and day 9 were tested by multispecies hematology instrument. E. The numbers of neutrophils, monocytes, eosinophils and lymphocytes in blood were tested by multispecies hematology instrument. F. Determination of ILC2s in lung tissue was tested by flow cytometry. G. The numbers of white blood cells (WBC), red blood cells (RBC) and quantities of hemoglobin (Hb), mean platelet volume (MPV), mean corpuscular hemoglobin concentration (MCHC) were tested by multispecies hematology instrument. (N =3 or 5; $*p < 0.05$, $**p < 0.01$, $***p < 0.001$, ns, not significant).

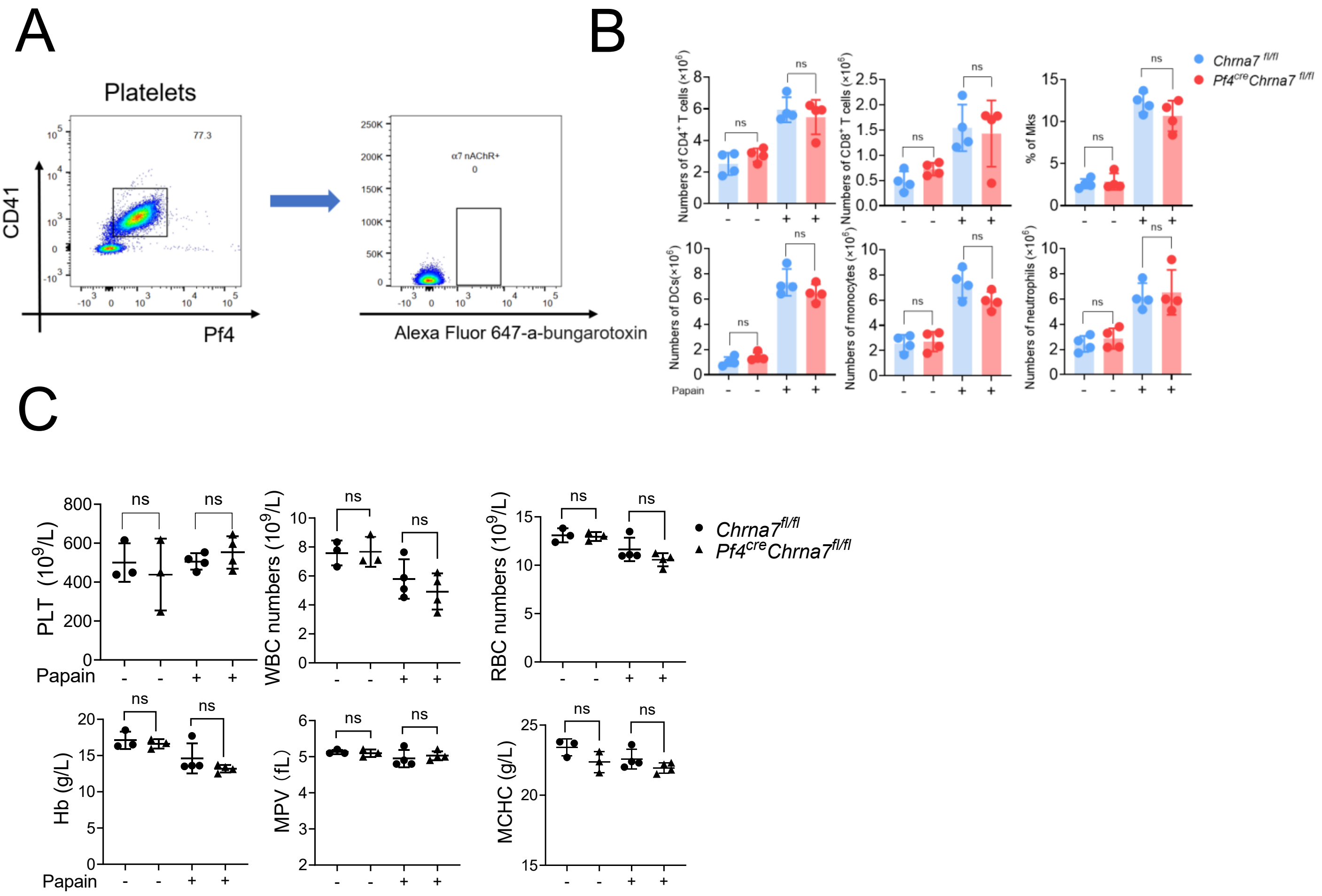

Figure S2 . Activation of α7nAChR on Mks doesn't affect T cells, B cells, DCs, monocytes, neutrophils, Mks in lung tissue and peripheral blood.

A. Expression of α7nAChR on platelets was tested by flow cytometry. B. The numbers of $CD4^+$ /$D8^+$ T cells, DCs, monocytes, neutrophils and percentage of Mks in lung tissue were determined by flow cytometry. C. The numbers of platelets, white blood cells (WBC), red blood cells (RBC) and quantities of hemoglobin (Hb), mean platelet volume (MPV), mean corpuscular hemoglobin concentration (MCHC) was tested by multispecies hematology instrument. (N =3-4; ns, not significant).

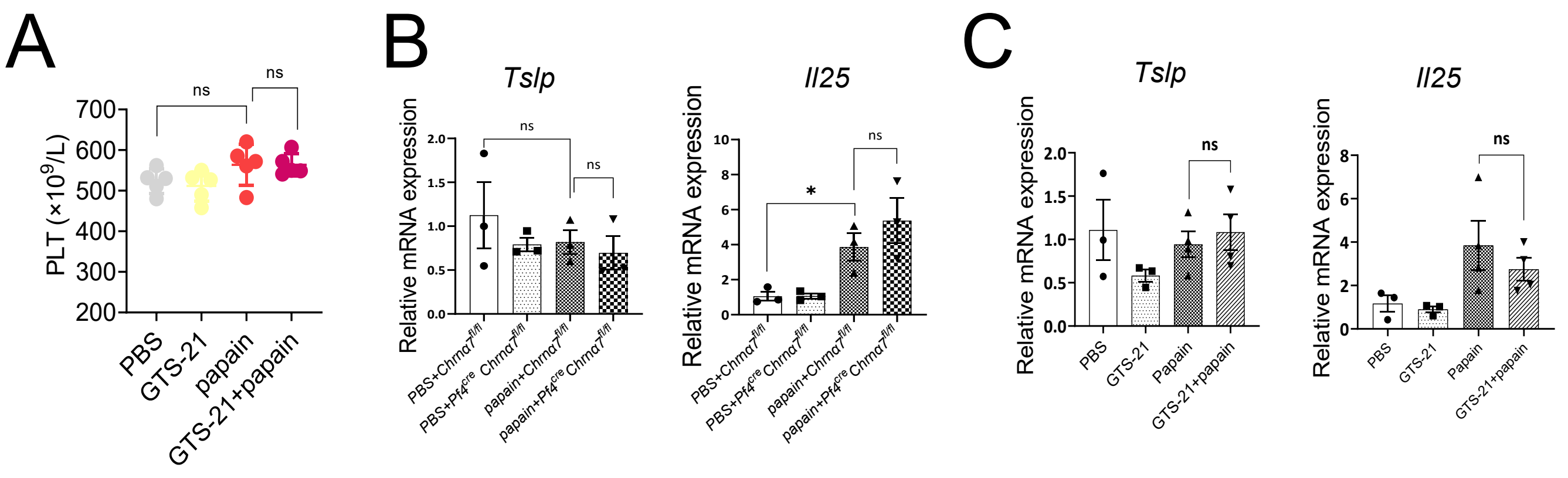

Figure S3 . Activation of α7nAChR on Mks doesn't affect platelet numbers in blood and the relative mRNA expression of Tslp and Il25 in lung tissue.

A. The number of platelets was tested by multispecies hematology instrument. B. The relative mRNA expression of *Tslp* and *Il25* in the lung tissue of *Chrna7*$^{fl/fl}$ and *Pf4*^{cre}*Chrna7*$^{fl/fl}$ mice under papain stimulation. C. The relative mRNA expression of *Tslp* and *Il25* in the lung tissue of WT mice under GTS-21 and papain stimulation. (N =3-5; *$p < 0.05$, ns, not significant).

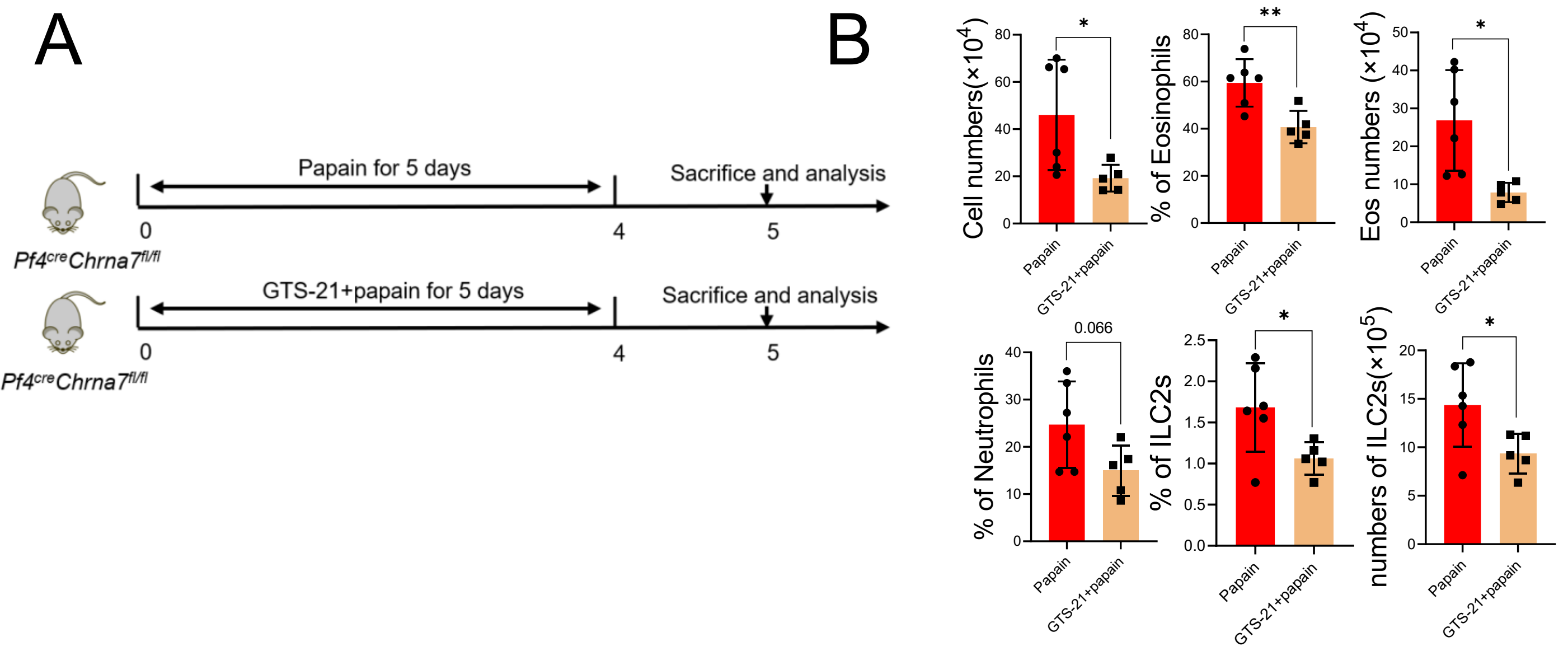

Figure S4. GTS-21 fails to amplify airway inflammation in *Pf4creChrna7fl/fl* mice.
A. Schematic diagram of GTS-21 intervention on papain-induced allergic airway inflammation model in *Pf4creChrna7fl/fl* mice. B. The percentage and numbers of inflammatory cells in BALF and lung were tested by flow cytometry. (N =5-6; **p* < 0.05, ***p* < 0.01).

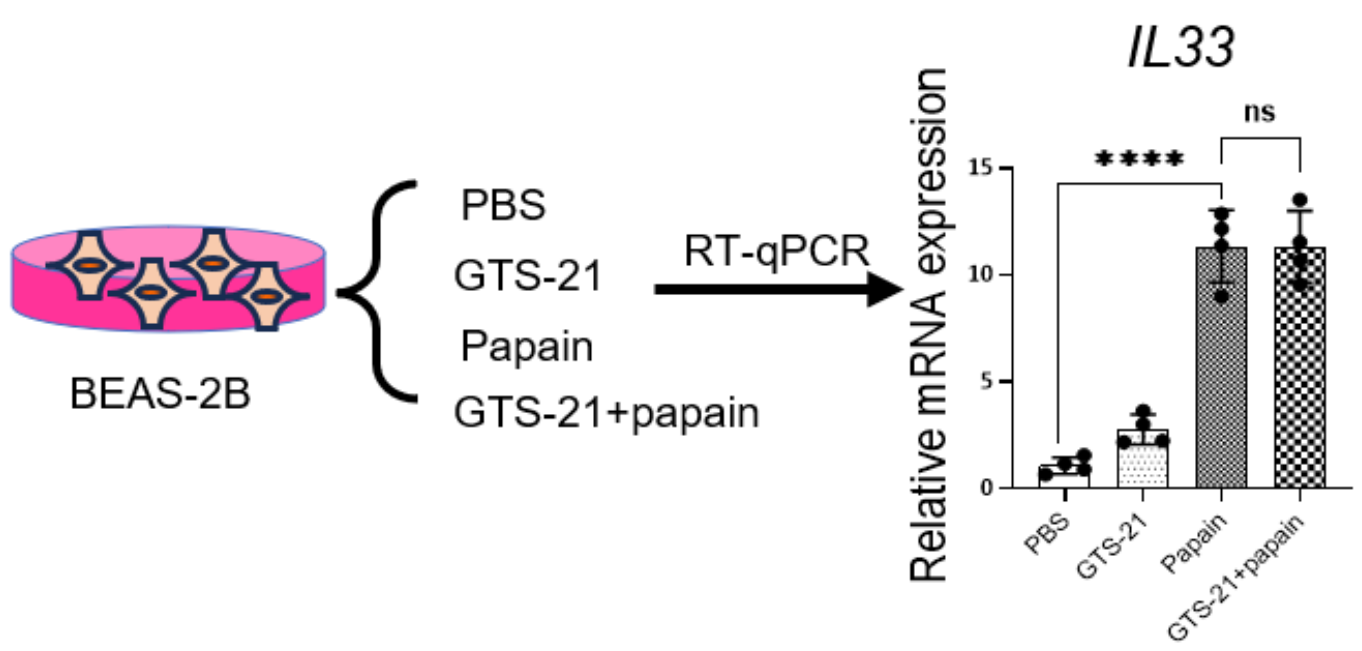

Figure S5. GTS-21 doesn’t affect the expression of *IL33* in BEAS-2B.
The relative mRNA expression of *IL33* in BEAS-2B treated with GTS-21 and papain tested by RT-qPCR. (N =4; ****p < 0.0001, ns, not significant).

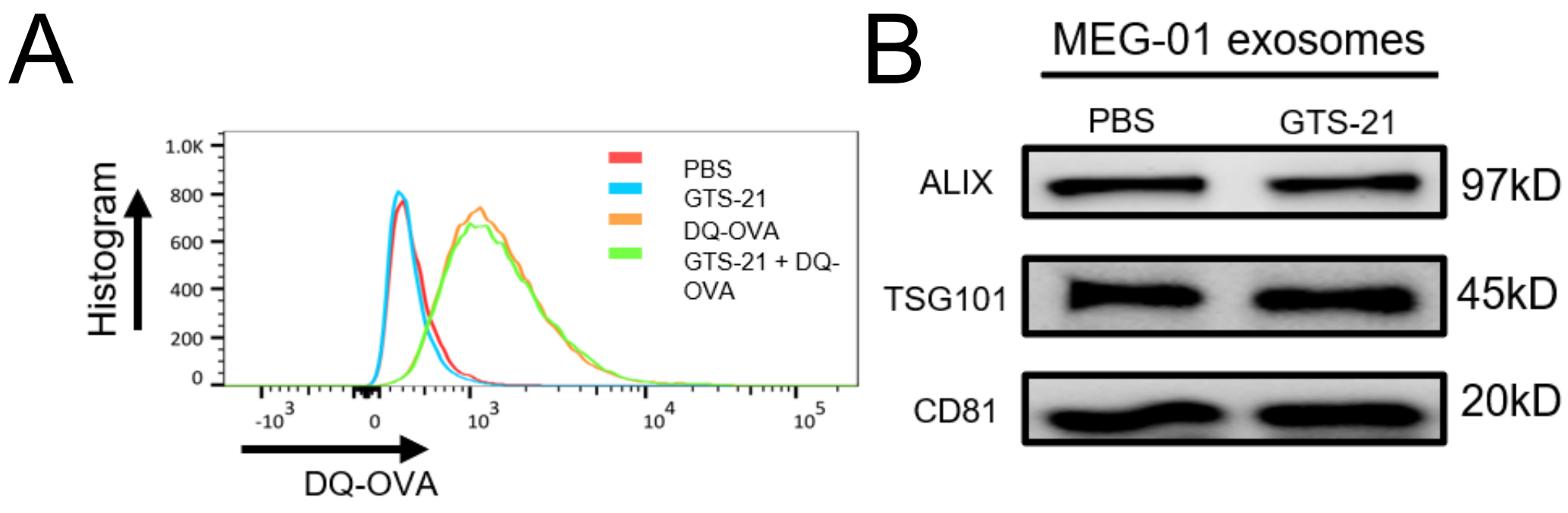

Figure S6. GTS-21 doesn’t affect the internalization of DQ-OVA and exosomes release in MEG-01.
A. Analysis for DQ-positive MEG-01 cells by flow cytometry. B. exosomes released from MEG-01 was obtained by ultracentrifugation and tested by western blot.
