# Supplementary material for: Activation of lung megakaryocyte α7 nAChR exacerbates allergic airway inflammation via IL-33/p38 MAPK signaling
Source: JCI Insight. 2026 Sep 8;11(17):e202201. doi: 10.1172/jci.insight.202201 (PMC13564082; doi:10.1172/jci.insight.202201)

Figure 7B (the original immunoblot images were subjected to contrast stretching)

IL-33

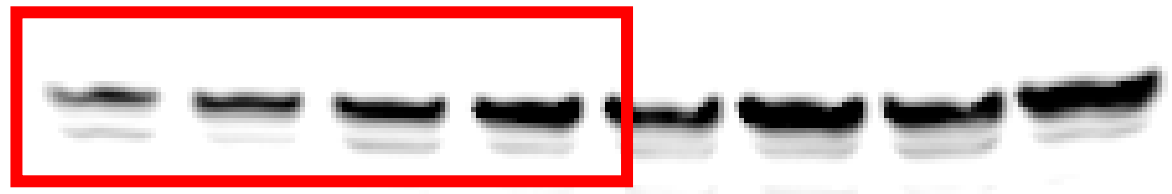

β-actin

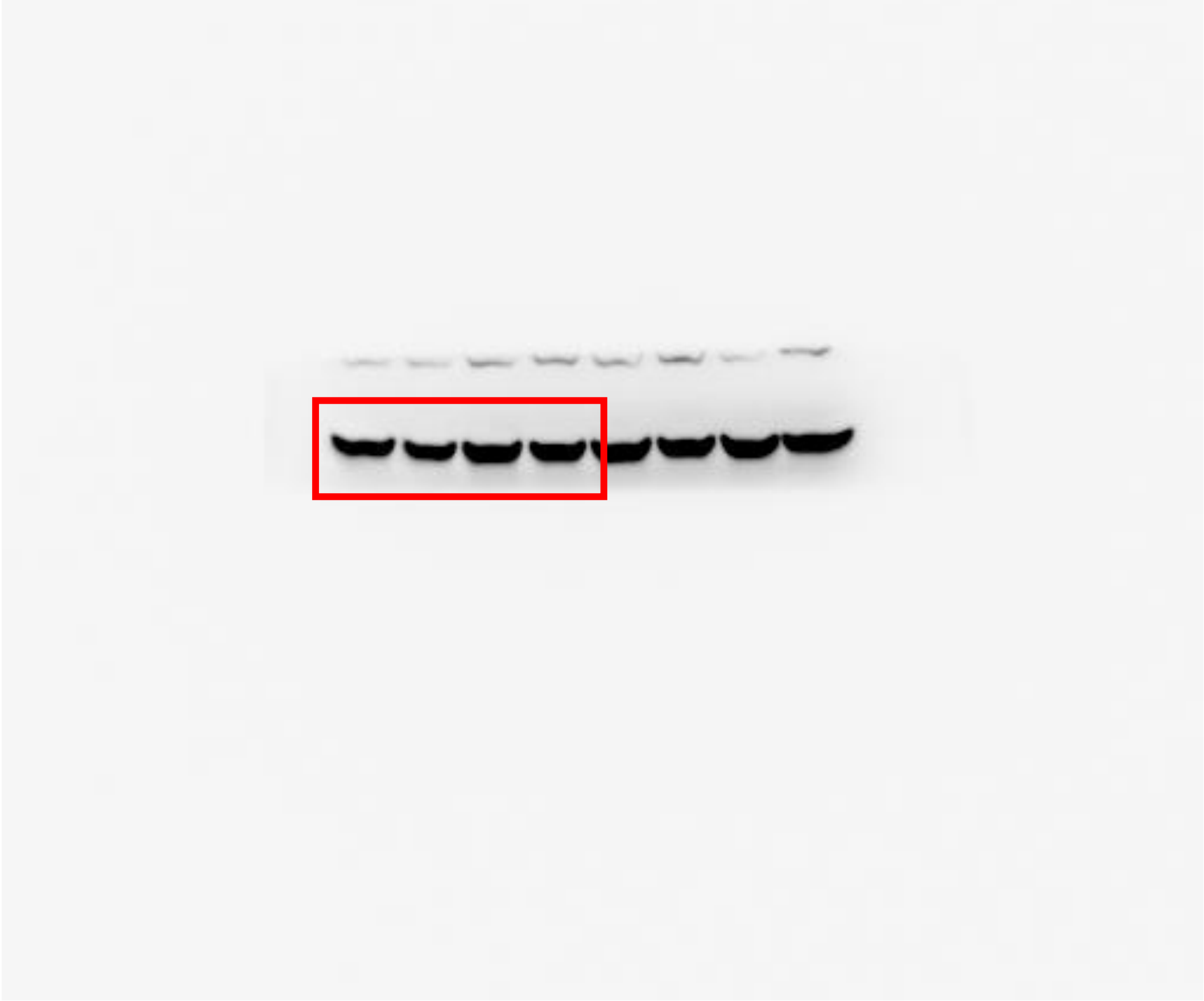

Figure 7C

IL-33

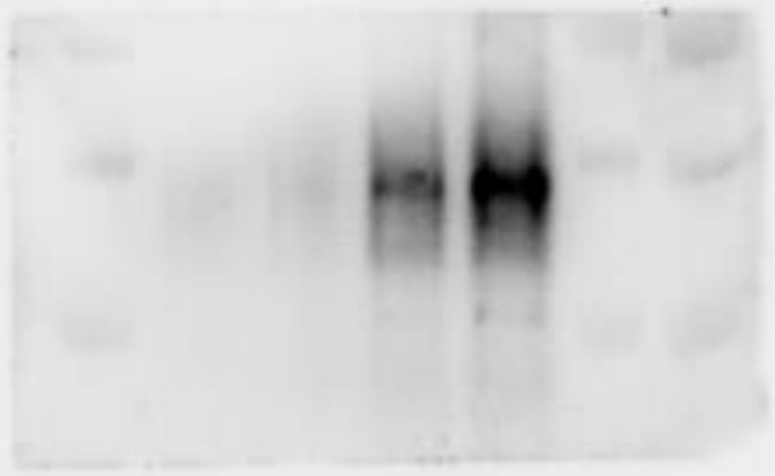

GAPDH

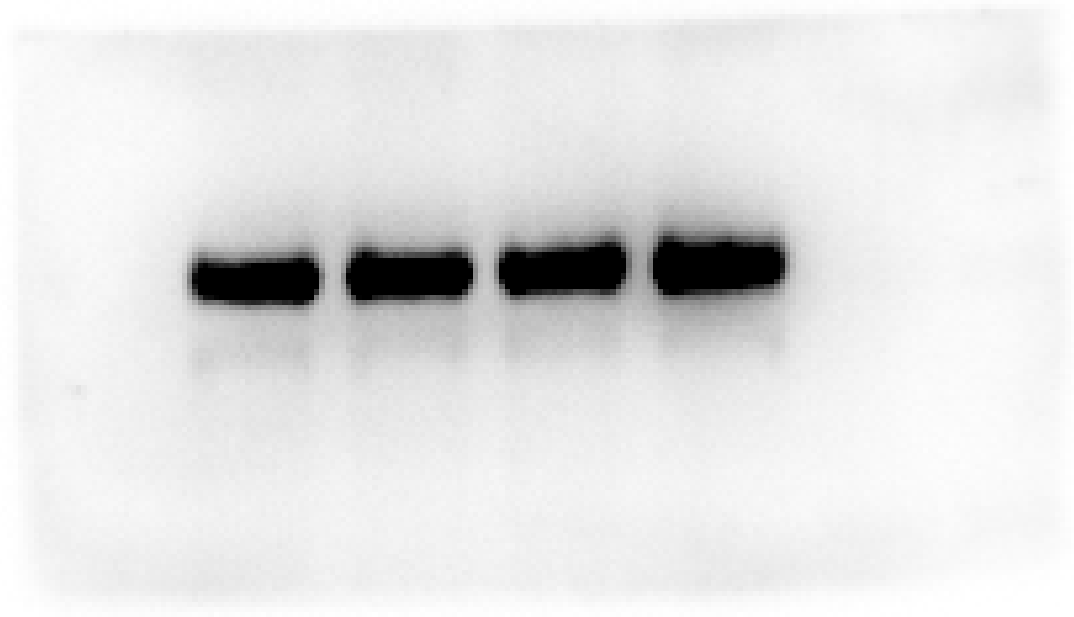

Figure 7F

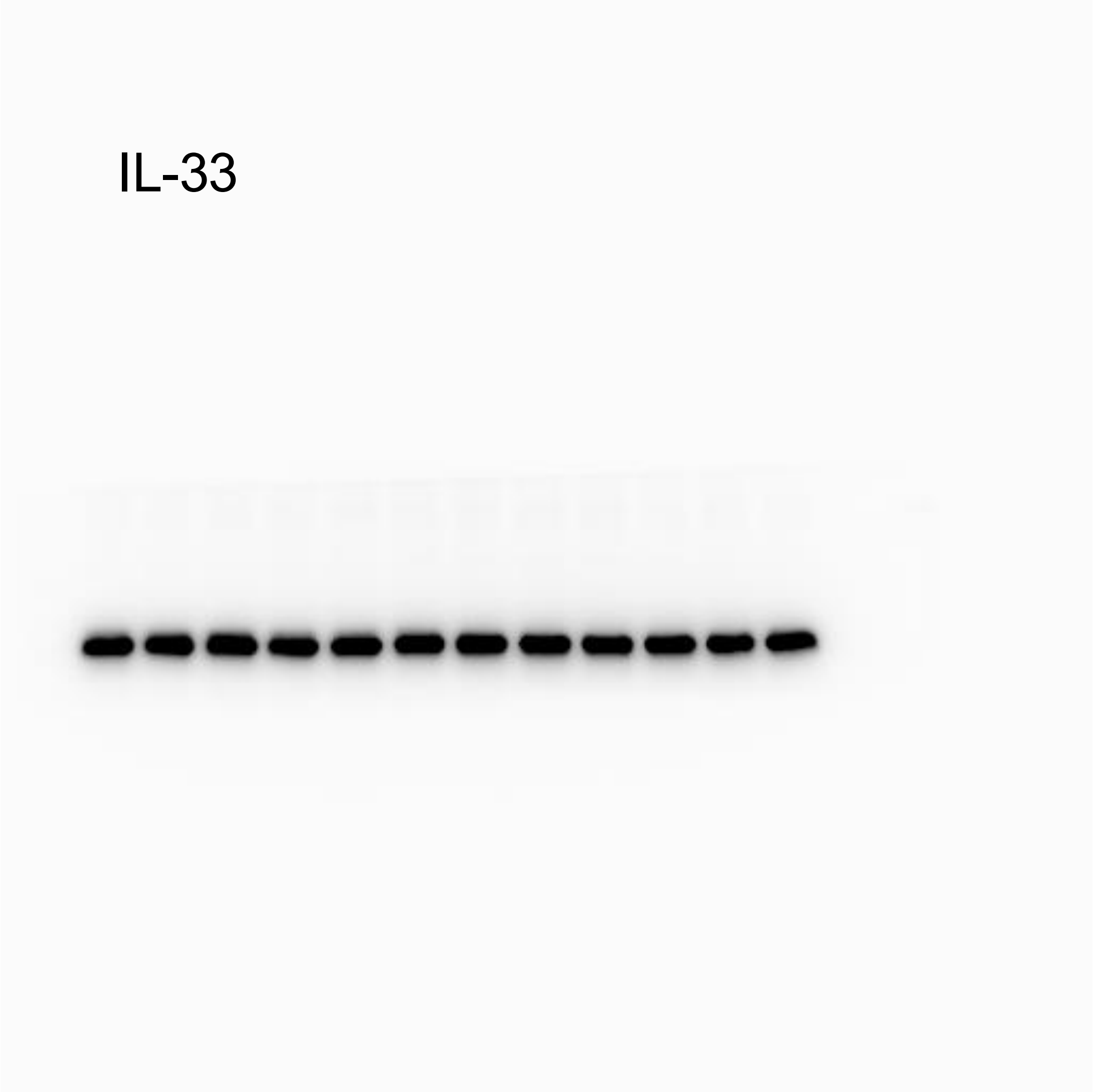

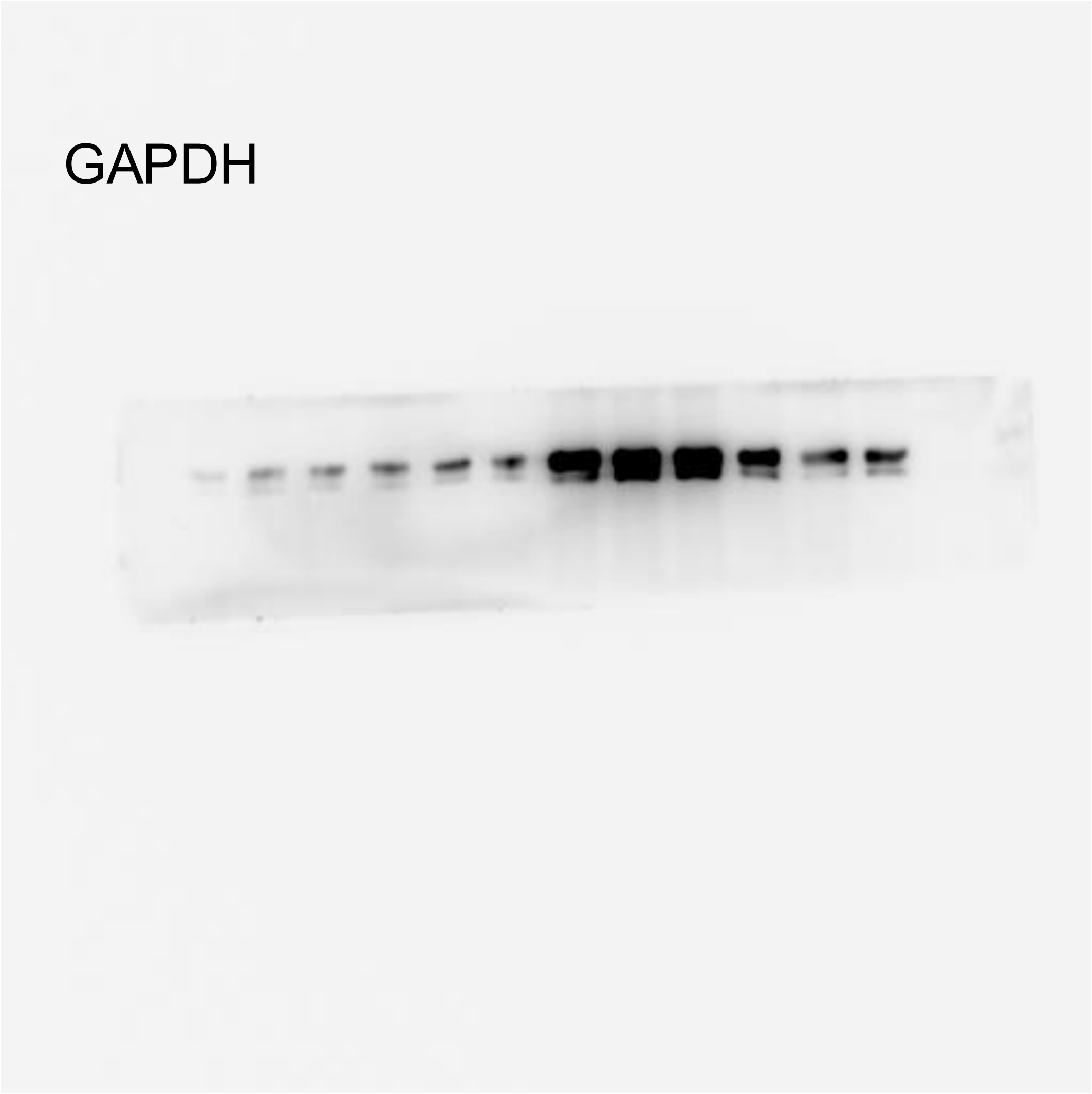

Figure 7F

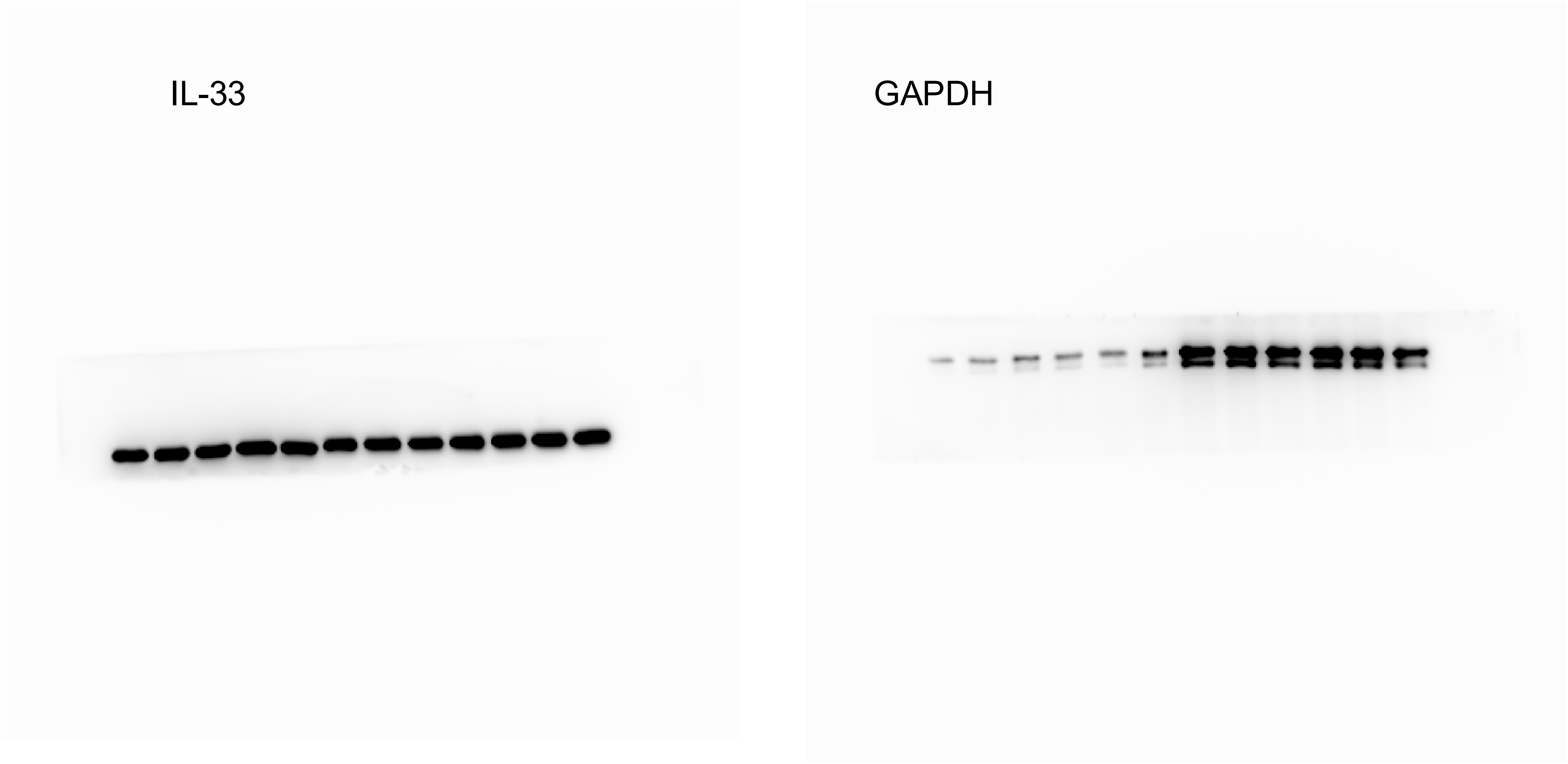

Figure 8A

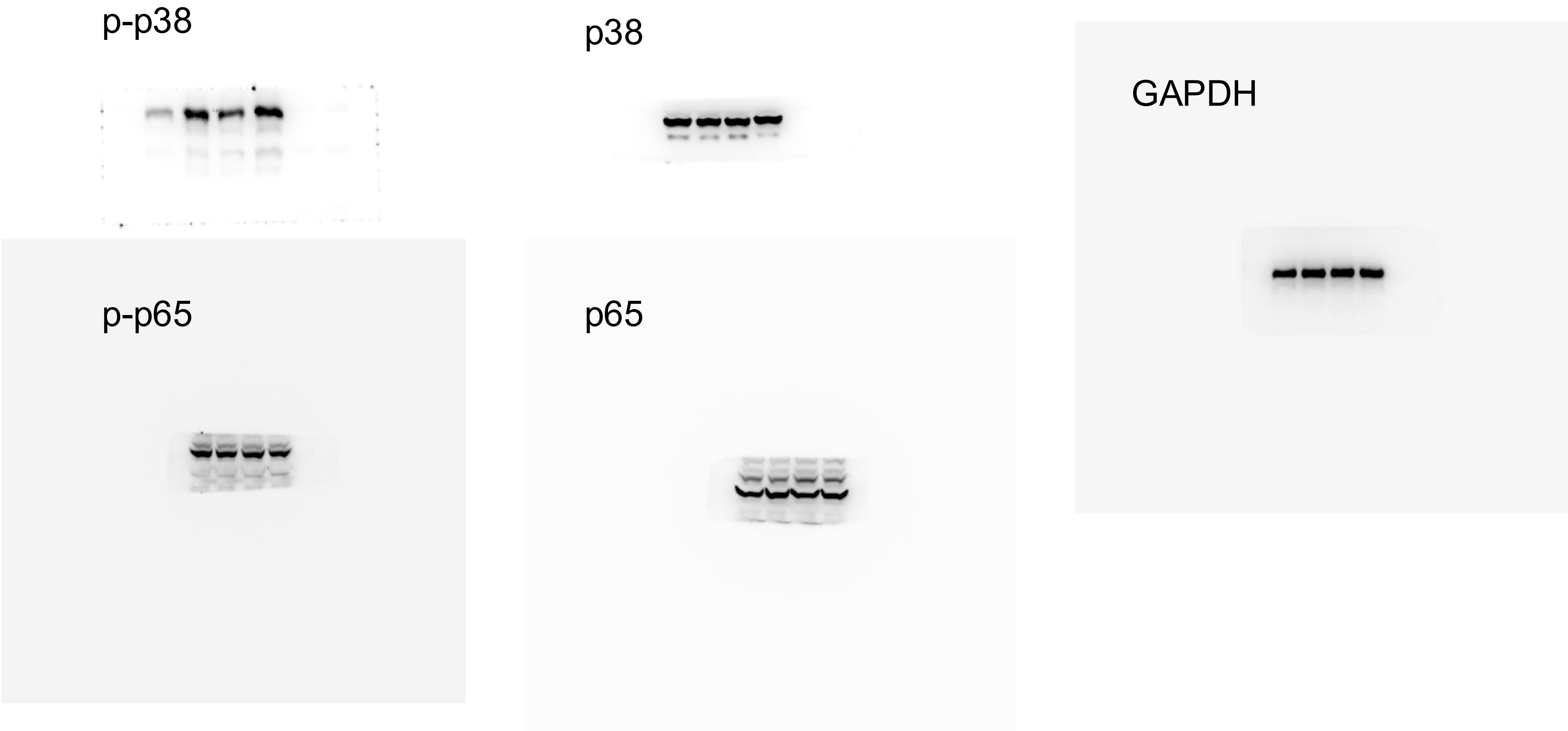

Figure 8G

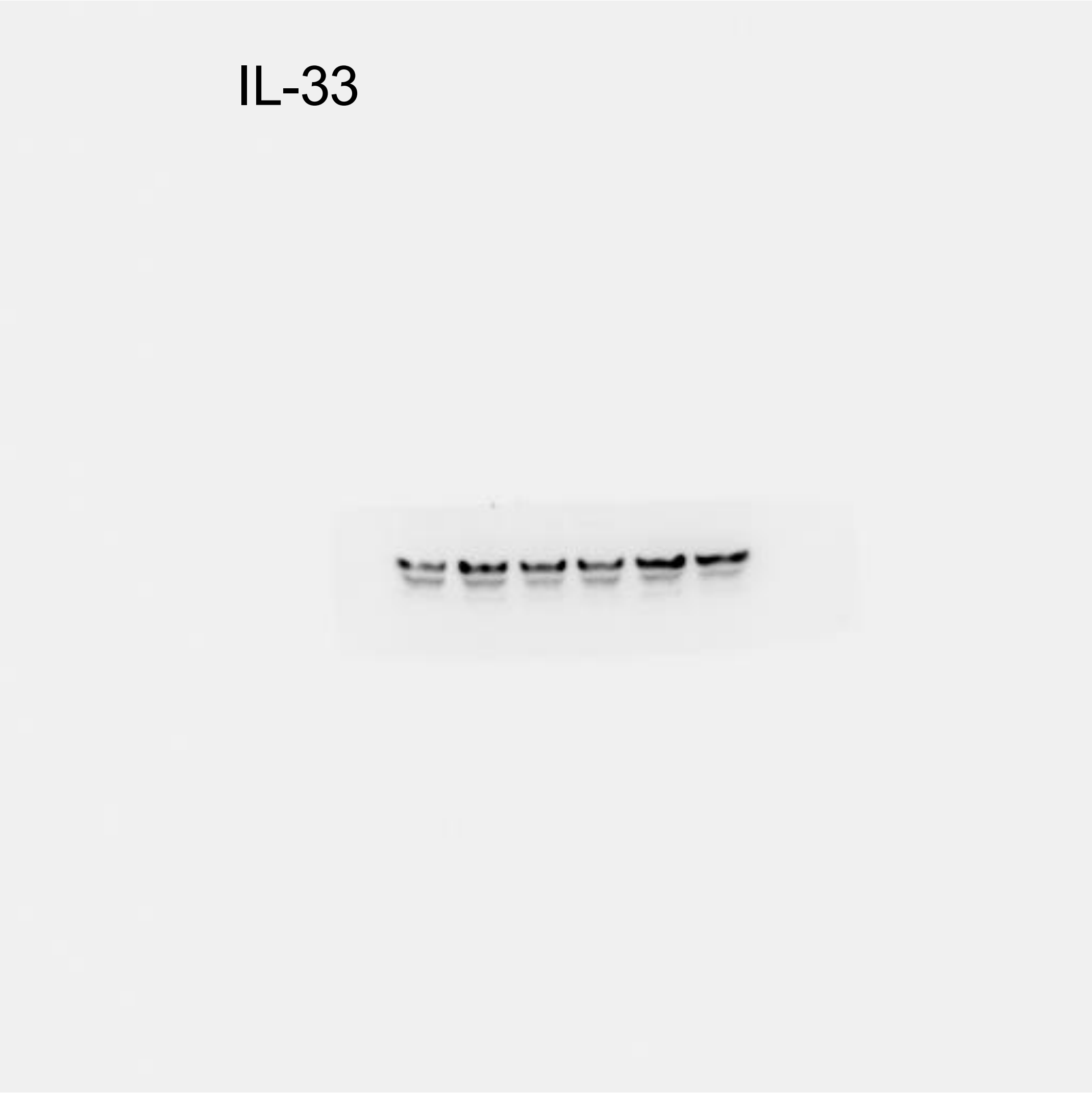

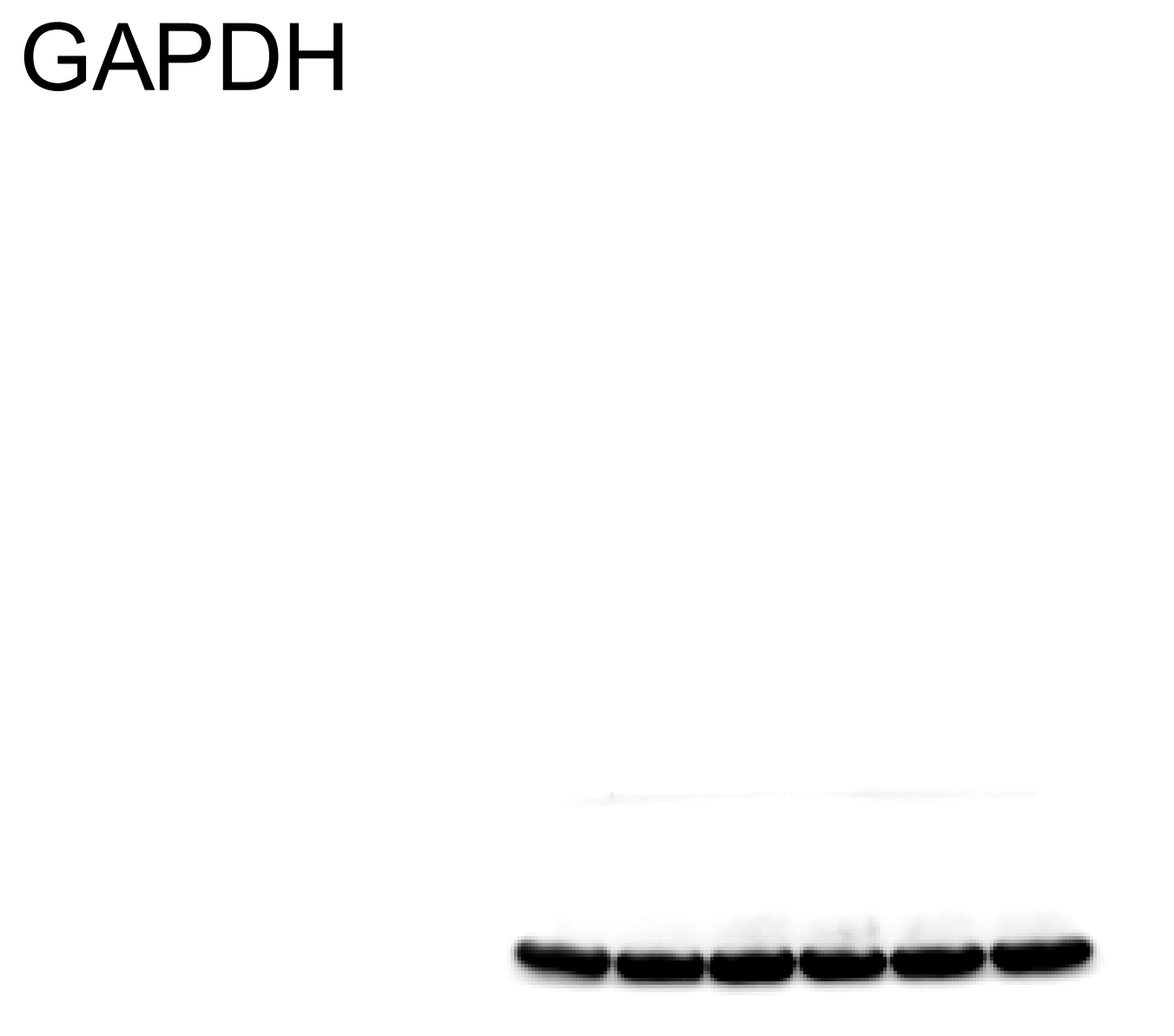

Figure S6B (the original immunoblot images were subjected to contrast stretching)

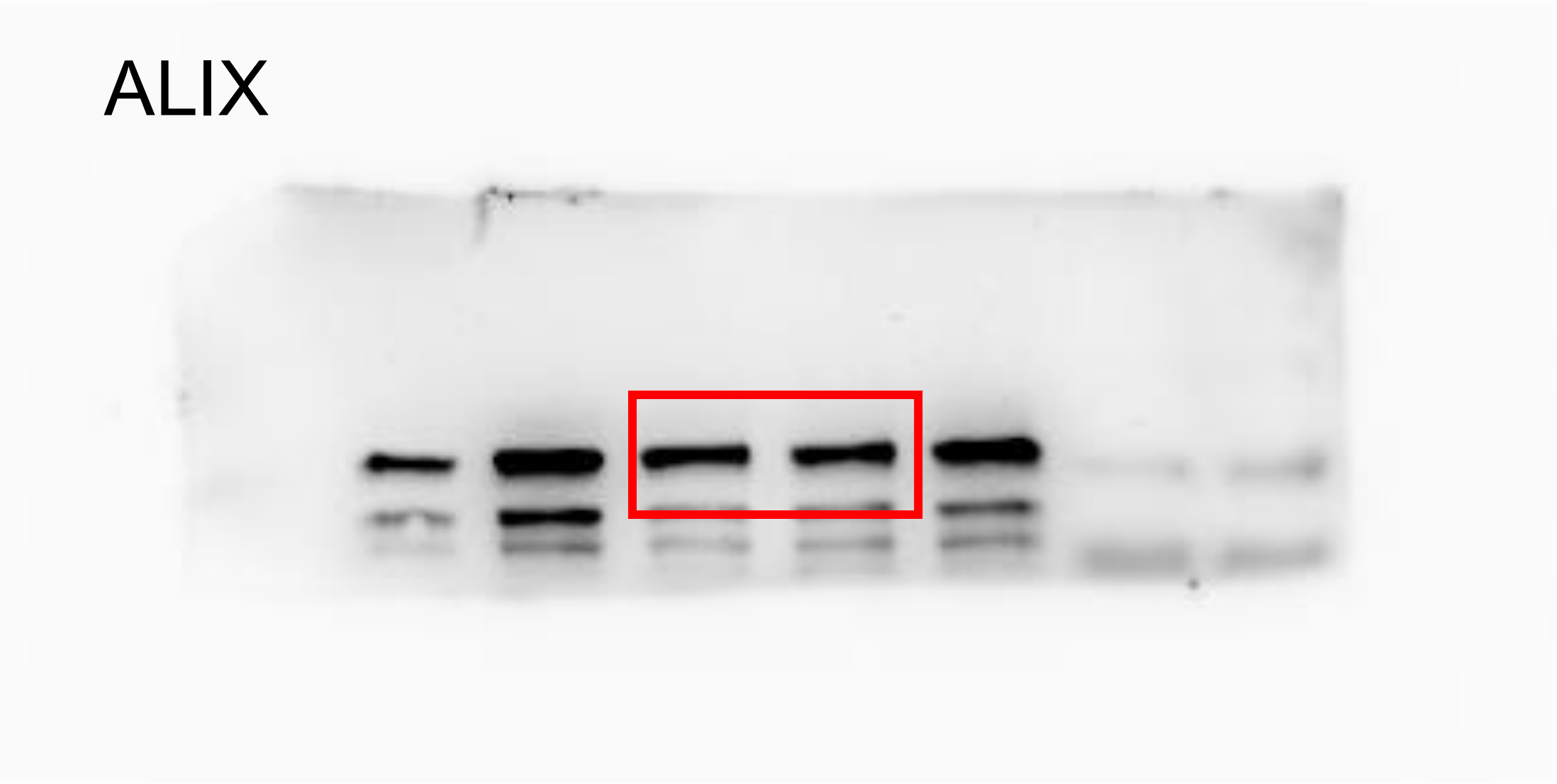

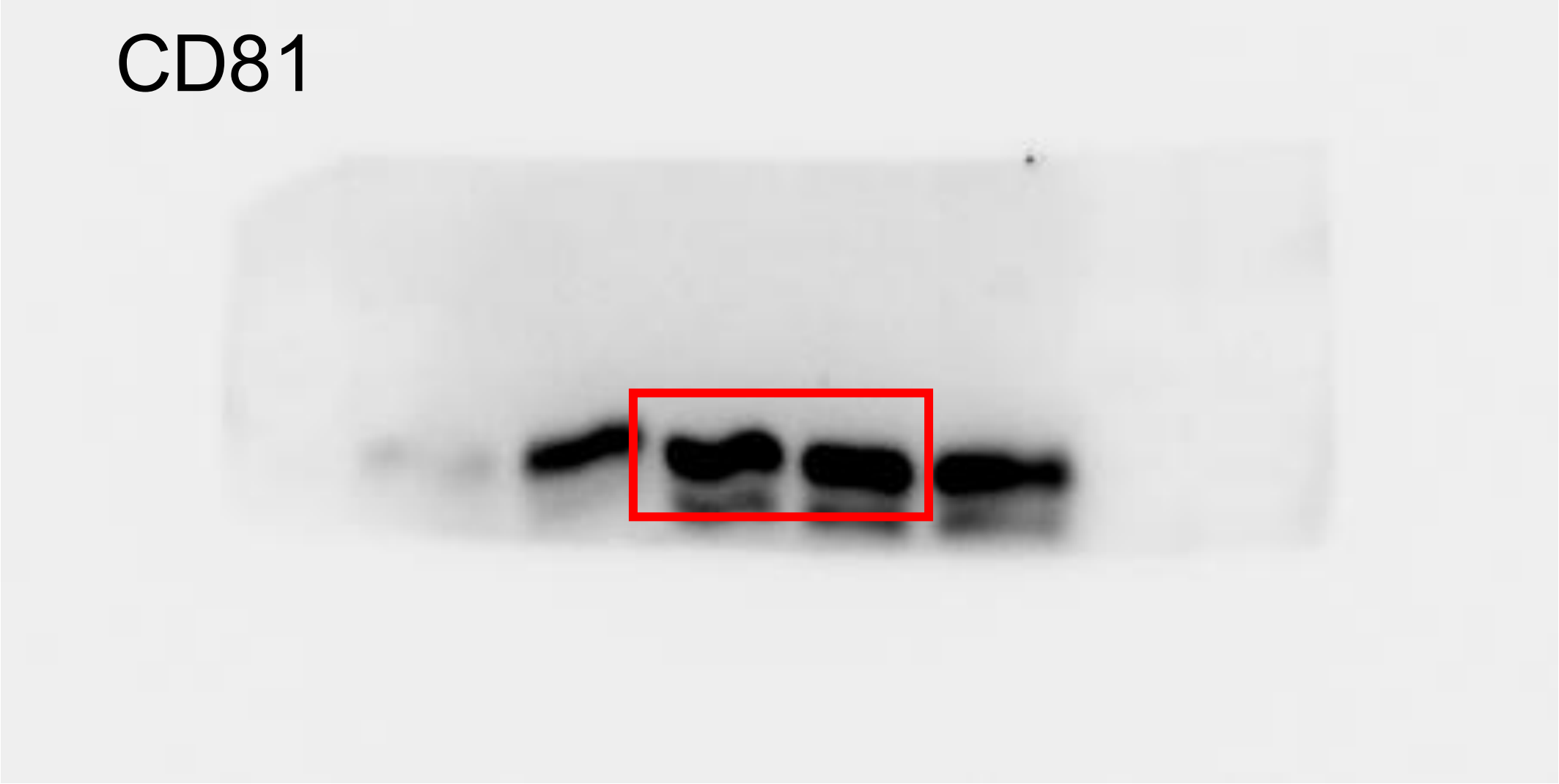

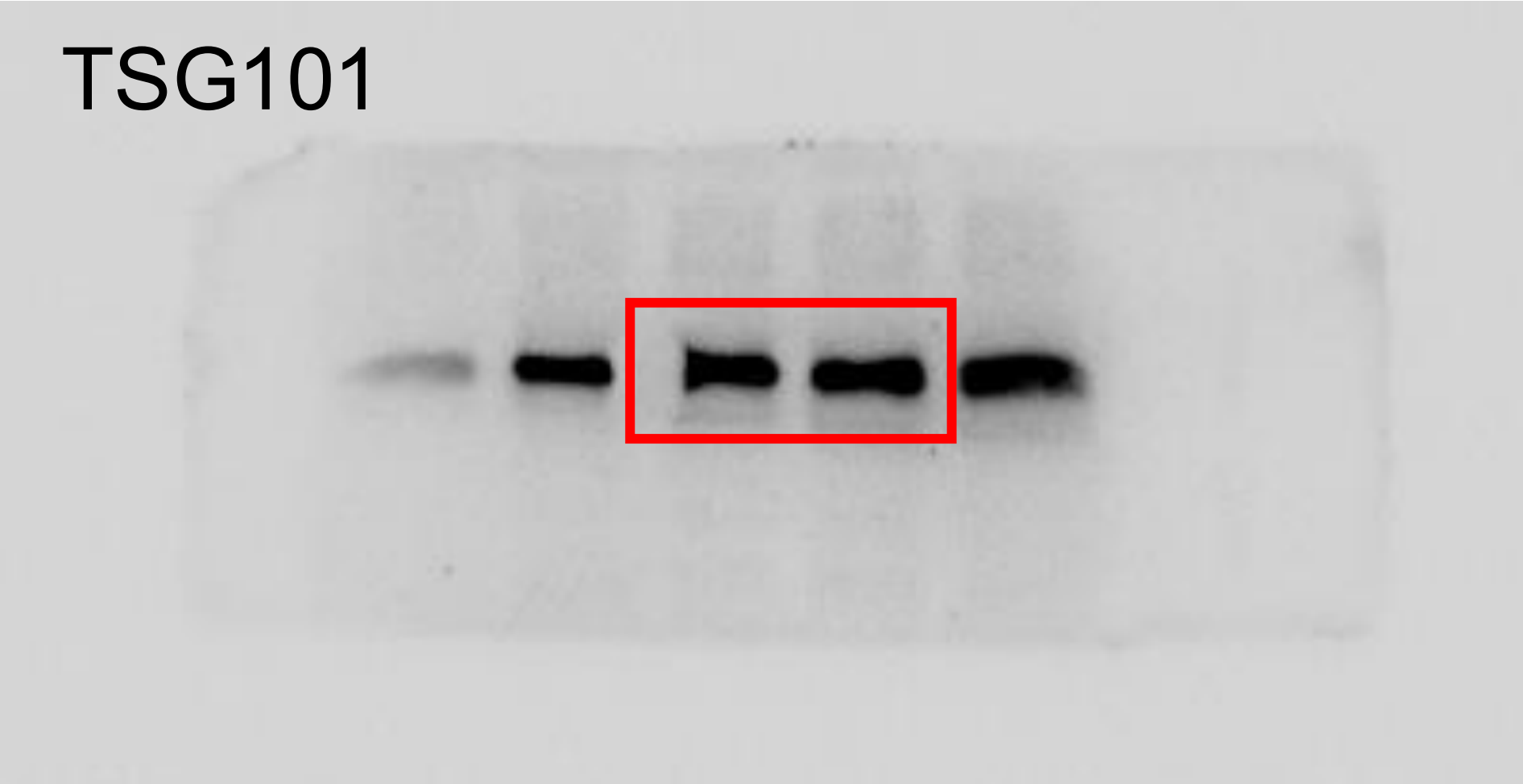

Supplement: Supplementary file 2 [file jciinsight-11-202201-s276.pdf]
